# Signals and Machinery for Mycorrhizae and Cereal and Oilseed Interactions towards Improved Tolerance to Environmental Stresses

**DOI:** 10.3390/plants13060826

**Published:** 2024-03-13

**Authors:** Aiman Slimani, Mohamed Ait-El-Mokhtar, Raja Ben-Laouane, Abderrahim Boutasknit, Mohamed Anli, El Faiza Abouraicha, Khalid Oufdou, Abdelilah Meddich, Marouane Baslam

**Affiliations:** 1 Centre d’Agrobiotechnologie et Bioingénierie, Unité de Recherche Labellisée CNRST (Centre AgroBiotech-URL-CNRST-05), Cadi Ayyad University, Marrakesh 40000, Morocco; 2 Laboratory of Agro-Food, Biotechnologies and Valorization of Plant Bioresources (AGROBIOVAL), Department of Biology, Faculty of Science Semlalia, Cadi Ayyad University, Marrakesh 40000, Morocco; 3 Laboratory of Microbial Biotechnologies, Agrosciences, and Environment, Department of Biology, Faculty of Science Semlalia, Cadi Ayyad University, Marrakesh 40000, Morocco; 4 Laboratory of Biochemistry, Environment & Agri-Food URAC 36, Department of Biology, Faculty of Science and Techniques—Mohammedia, Hassan II University, Mohammedia 28800, Morocco; 5 Laboratory of Environment and Health, Department of Biology, Faculty of Science and Techniques, Errachidia 52000, Morocco; 6 Multidisciplinary Faculty of Nador, Mohammed First University, Nador 62700, Morocco; 7 Department of Life, Earth and Environmental Sciences, University of Comoros, Patsy University Center, Moroni 269, Comoros; 8 Higher Institute of Nursing and Health Techniques (ISPITS), Essaouira 44000, Morocco; 9 AgroBiosciences Program, College of Agriculture and Environmental Sciences, University Mohammed VI Polytechnic (UM6P), Ben Guerir 43150, Morocco; 10 GrowSmart, Seoul 03129, Republic of Korea

**Keywords:** mycorrhizal symbiosis, cereal crops, oilseed crops, stress mitigation, tolerance, (a)biotic stresses, molecular drivers, mutualism

## Abstract

In the quest for sustainable agricultural practices, there arises an urgent need for alternative solutions to mineral fertilizers and pesticides, aiming to diminish the environmental footprint of farming. Arbuscular mycorrhizal fungi (AMF) emerge as a promising avenue, bestowing plants with heightened nutrient absorption capabilities while alleviating plant stress. Cereal and oilseed crops benefit from this association in a number of ways, including improved growth fitness, nutrient uptake, and tolerance to environmental stresses. Understanding the molecular mechanisms shaping the impact of AMF on these crops offers encouraging prospects for a more efficient use of these beneficial microorganisms to mitigate climate change-related stressors on plant functioning and productivity. An increased number of studies highlighted the boosting effect of AMF on grain and oil crops’ tolerance to (a)biotic stresses while limited ones investigated the molecular aspects orchestrating the different involved mechanisms. This review gives an extensive overview of the different strategies initiated by mycorrhizal cereal and oilseed plants to manage the deleterious effects of environmental stress. We also discuss the molecular drivers and mechanistic concepts to unveil the molecular machinery triggered by AMF to alleviate the tolerance of these crops to stressors.

## 1. Introduction

Cereals and oilseeds, and their derived products, play significant roles in the daily diets of people worldwide. As the global population continues to expand, the demand for these crops and their products is on the rise [1,2]. However, numerous factors, including climate change, deteriorating soil health, land contamination, and agricultural practices, pose serious threats to agricultural productivity. The escalating frequency of extreme weather events, driven by climate change, further imperils crop output [3]. A single or multiple combination of abiotic stressors, such as heat, salt, drought, heavy metal toxicity, and soil depletion, along with biotic factors, present primary constraints on crop production and quality. Environmental challenges, including reduced nutrient uptake and biosynthetic ability, can hinder plant performance. Moreover, alteration in stress conditions can impact signaling pathways, protein synthesis, and gene expression, leading to the activation of stress-responsive transcription factors. This enables the adaptation of downstream responses required to support a successful defense against particular constraints [4,5]. These changes have profound implications for global agricultural sustainability and food security, emphasizing the pressing need for innovative solutions [6,7].

In the intricate soil ecosystem, the rhizosphere is a nexus of microbial activity where both fungi and bacteria reside, each playing a pivotal role in plant health and growth. Arbuscular mycorrhizal fungi (AMF) are well documented for their symbiotic relationships with plants, enhancing nutrient uptake, particularly phosphorus (P), and bolstering plant defense mechanisms. Arbuscular mycorrhizal (AM) fungi, such as *Rhizophagus irregularis*, *Funneliformis mosseae*, and *Glomus versiforme*, are integral components of the soil microbiome and establish mutualistic relationships with ca. 90% of agricultural plants, particularly cereals and oilseeds [8]. These obligate symbionts rely on the host carbon (C) compounds—up to 20% of plant-fixed C—, contributing significantly to their survival [9]. In return, through specialized structures called arbuscules, the fungi enhance the host water and nutrient supply, particularly P and nitrogen (N) [10]. AMF demonstrate the potential to alleviate the negative effects of abiotic stresses by modifying physiological responses, enhancing nutrient absorption, facilitating osmotic adjustment, and reducing oxidative damage [11,12,13,14]. They also assist host plants in maintaining an appropriate ion balance under these extreme conditions [15,16], as evidenced by the significantly increased shoot biomass of AM-symbiotic plants compared to non-colonized plants [17]. In addition to mitigating abiotic stress, AMF symbiosis can aid host plants in combating biotic stresses, particularly pathogen infections. The influence of AMF on root microbiome abundance and composition, alteration in root exudation, enhancement of host nutrition, and induction of the plant defense system contribute to the beneficial effects of AM symbiosis against pathogen infection [18,19]. In addition, the rhizosphere also teems with diverse bacterial populations that can stimulate plant growth through various mechanisms, such as nitrogen fixation, phytohormone production, and the solubilization of minerals. While both AMF and rhizosphere bacteria can have stimulatory effects on plants, their modes of action exhibit fundamental differences. AMF primarily facilitate the direct uptake of nutrients through extensive hyphal networks, whereas rhizosphere bacteria often modify the soil environment or plant physiology to promote growth. Moreover, the bacteria can also play a role in inducing systemic resistance to pathogens, similar to AMF, but through different signaling pathways and microbial interactions. Understanding these distinct yet potentially complementary roles of fungi and bacteria in the rhizosphere is crucial for harnessing their full potential in agricultural systems, leading to more sustainable crop production strategies.

The AMF-induced responses under environmental stresses are controlled by tight and complicated molecular mechanisms connecting all cellular levels’ functioning [15,20].

A deeper understanding of the molecular mechanisms governing the effects of AMF could lead to improved performances of cereal and oilseed crops under changing climate conditions. By influencing soil nutrient and C cycling and enhancing nutrient and water absorption and crop resistance to stress, mycorrhizal symbiosis remains a significant component of terrestrial ecosystems, with a pivotal role in shaping food systems under climate change scenarios. While the impact of (a)biotic stresses on plant species, including cereal and oilseed crops, has been extensively studied, there remains a notable gap in understanding the underlying molecular mechanisms and the ‘cross-talk’ among AMF, biotic and abiotic stressors, and cereal and oilseed crops. Our review addresses this critical gap by focusing on the molecular patterns that govern the interaction of AMF with these crops under environmental stresses, marking a significant advancement in environmentally friendly crop management strategies and sustainable agriculture under changing environments. This emphasis on the intricate molecular mechanisms underscores the originality and significance of our work, positioning it as an innovative contribution to the field. In this context, we emphasize the multifaceted benefits of AMF in promoting the tolerance of grain and oil crops to biotic and abiotic stress. Furthermore, we advocate for an exploration of the molecular pathways regulating mycorrhizal symbiosis-induced resilience in these crops to mitigate stressors. Finally, we propose avenues for future research to deepen our understanding of the signals and molecular machinery underlying the interaction between AMF and plants, towards the advancement of smart, resilient, and sustainable agriculture.

## 2. Building Resilience: Harnessing Mycorrhizal Symbiosis for Enhanced (a)biotic Stress Tolerance in Cereal and Oilseed Crops

Environmental stressors inflict substantial economic losses by diminishing the yield and quality in cereal and oilseed crops [21,22,23]. Drought, salinity, and heavy metals (HMs) exposure are among the primary abiotic factors that pose a threat to the growth and development of these crops [24]. Water limitation often results in stomata closure, impacting CO_2_ efflux and photosynthesis. Similarly, salinity disrupts mineral uptake and increases osmotic pressure in soil solutions, while HMs alter Ca^2+^ channel activity, affecting metallic assimilation, transport, and metabolism [25,26]. AMF applications have been found to enhance plant responses to these stresses through various mechanisms, including improved nutrient uptake, production of phytohormones, osmotic adjustment, maintenance of homeostasis, oxygen radical scavenging, and antioxidants [25,26]. Additionally, cereal and oilseed crops face biotic stresses stemming from viruses, bacteria, fungi, and insect herbivores. However, AMF play a crucial role in mitigating these stresses by inducing disease resistance through the increased production of antioxidants and reinforcement of the defensive machinery [25,26]. Furthermore, AMF contribute to improvements in the nutrient profile, root system growth and architecture, photosynthetate levels, and alterations in the mycorrhizosphere microbial community [27].

Of note, the documented beneficial effects of AMF on plant fitness and environmental stress tolerance underscore their significance in ecological contexts. However, it is crucial to acknowledge the susceptibility of these microorganisms to climate change-related stresses, as observed alterations in AMF biodiversity, abundance, and key developmental stages in response to abiotic stresses have significant ecological implications. Abiotic stresses have been shown to exert notable impacts on AMF, affecting critical developmental stages such as germination, colonization, extraradical hyphal elongation, and sporulation, leading to morphological adaptations in response to changing environmental conditions [28]. Specifically, aridity has been associated with a decline in spore production and AMF species richness. Despite these challenges, the presence of resilient spores and rapid mycelial growth have emerged as potential factors contributing to the performance of AMF, particularly in dry conditions, thus highlighting the adaptive resilience of these microorganisms to environmental changes [29]. These insights reveal the intricate interplay between AMF and climate change-related stresses, shedding light on their adaptive capabilities in the face of evolving environmental conditions, and emphasizing the need to comprehensively understand and monitor the responses of these crucial symbiotic microorganisms to environmental alterations.

### 2.1. Cereal Mycorrhizal Responses to Stressed Environments

#### 2.1.1. Cereal Mycorrhizal Responses to Abiotic Stresses

AMF engage in symbiotic interactions with a majority of cereal plants, aiding them in combating the adverse effects of various stresses. This section presents a summary of AMF evaluated for their roles in mitigating abiotic stress in cereal crops.

AMF application has demonstrated efficacy in alleviating the detrimental impacts of drought, a significant abiotic stressor for cereal crops. Studies on maize and wheat have revealed that AMF mitigate drought stress by enhancing water status and uptake, primarily through improved water use efficiency (WUE) [30,31,32,33]. *Rhizoglomus intraradices* and *Rhizophagus irregularis* maintained a higher WUE in maize and wheat, respectively, attributed in part to their modulation of aquaporin gene expression, facilitating water and small neutral molecule passage across cell membranes in host plants [34]. Moreover, AMF establish an extensive hyphal network with the plant root system, particularly fine root hairs, enhancing accessibility to a larger soil surface area and water retention. Bernardo et al. [35] reported that *Funneliformis mosseae*-inoculated wheat exhibited improved WUE under water limitation, while Kamali and Mehraban [36] observed similar results in sorghum inoculated with *Glomus mosseae*. Furthermore, AMF contribute to enhanced N and P contents in maize and wheat by exploring solution-filled soil pores inaccessible to plant roots and facilitating nutrient uptake [34,37]. Through the enhancement of alkaline and acid phosphatase levels, AMF contribute to improved P availability and assimilation [28,30].

In addition to the nutrient exchange, phytohormones play a crucial role in regulating AM interactions at later stages [38]. Analysis of AM symbiosis regulation by phytohormones has revealed a complex pattern of modified hormonal contents and/or altered responses to hormones in mycorrhizal plants under drought conditions. Across a wide range of cereal/fungal species and experimental setups, auxins (IAA), abscisic acid (ABA), and brassinosteroids (BRs) have been identified as positive regulators of the AM symbiosis, while gibberellins (GAs) and salicylic acid (SA) have been described as negative regulators of the interaction [39,40,41]. Chareesri et al. [42] demonstrated that AMF inoculation led to enhanced ABA and IAA production in rice. IAA stimulates root development, while ABA, when transported to the leaves, acts as a regulator of the stomatal aperture.

AMF confer an advantage by enhancing the photosynthetic machinery under water limitation, positively regulating Calvin cycle enzymes and reactive oxygen species (ROS) scavenging capabilities. Studies conducted in sorghum, maize, and wheat have shown that mycorrhizae regulate stomatal aperture, increase stomatal density, and enhance the maximum quantum efficiency of photosystem I (PSI) and PSII [43,44,45,46]. Additionally, AMF colonization has been found to increase the chlorophyll content and photosynthate production, resulting in increased plant biomass [46,47]. It has been suggested that the C used by symbiotic AMF may be compensated by higher photosynthetic rates, as fungal metabolism creates a strong C sink, preventing photosynthate accumulation and photosynthesis down-regulation. Furthermore, AMF may induce leaf CO_2_ absorption, providing more area for solar assimilation [48,49].

AMF intervene in stress mitigation by enhancing plants’ osmotic adjustment through the accumulation of osmoregulators such as soluble proteins, sugars, proline, and glycine betaine. Studies have reported an increase in sugars and proline contents in finger millet plants inoculated with *R. intraradices* [50] and amino acids, sugars, proline, and glycine betaine levels in maize inoculated with *G. versiforme* [51]. Moreover, by modulating the antioxidant defense pathway, AMF symbiosis can improve ROS scavenging under stressful conditions. Research has demonstrated that AMF reinforce the antioxidant defense machinery, leading to reduced electrolyte leakage (EL), hydrogen peroxide (H_2_O_2_), and malondialdehyde (MDA) content, while boosting enzyme activity such as superoxide dismutase (SOD), polyphenol oxidase (PPO), and peroxidase (POX) in cereal crops under water deficit [48,49].

Salt stress poses a significant environmental constraint for cereal crops, leading to ionic toxicity and osmotic stress, ultimately impeding plant growth and development. However, mycorrhizae have been shown to mitigate salt-induced effects on plants. Studies on maize, wheat, and rice have demonstrated that the application of AMF mitigates the adverse effects of salinity, offering valuable insights into its potential to counteract salinity-induced harm [52,53,54]. The inoculation with *F. mosseae*, *G. mosseae*, or *R. irregularis* improves WUE, physiological traits, nutritional uptake, defensive machinery, and ROS scavenging abilities under salt-stressed conditions [55,56,57]. AMF promote the reduction in Na^+^ and Cl^−^ ions uptake, contributing to maintaining vacuolar membrane stability and facilitating the sequestration of Na^+^ into plants’ vacuoles. This mechanism has been reported in several cereal crops, including wheat [58,59], maize [56,60,61,62], rice [63,64,65], and sorghum [63,66,67]. Selvakumar et al. [61] found higher K^+^/Na^+^ and *ZmAKT2* (a phloem-expressing K^+^ channel), *ZmSOS1* (with ability to extrude Na^+^ and control xylem loading for a long-distance Na^+^ transport), and *ZmSKOR* (involved in the translocation of K^+^ towards shoots through xylem) gene expression in maize root inoculated with *Gigaspora margarita* or *Claroideoglomus lamellosum*. AMF have been observed to facilitate partial shoot Na^+^ translocation/efflux from the cytosol to vacuoles, resulting in reduced Na^+^ content in root tissues. Furthermore, the application of AMF has been found to enhance the properties of saline soils. Krishnamoorthy et al. [52] reported that cereal plants treated with *R. intraradices* exhibited greater easily extractable glomalin-related soil protein (EE-GRSP) content under saline conditions. The secretion of GRSP, a hydrophobic glycoprotein produced by the fungal extra-matricial mycelium, allows AMF to act as soil-binding agents, stabilizing soil aggregation and promoting soil fertility [68]. Under saline conditions, AMF have been shown to increase soil Olsen-P, enhance telluric microorganisms, and boost microbial enzymes, such as dehydrogenases and alkaline phosphatases. Additionally, mycorrhizae function as P-activating agents, accelerating the transformation of P to bio-available forms through the action of enzymes, including phosphatases. Chandra et al. [66] showed similar trends in salt-treated sorghum plants inoculated with *F. mosseae* or *F. geosporum*.

HMs present a significant constraint for cereal crops when present in excess. Mycorrhizae have demonstrated the potential to promote the phytoremediation of HMs by reducing their assimilation. The effectiveness of AMF-assisted HMs phytoremediation is contingent upon the specific mycorrhizal fungi species. Hao et al. [69] observed a decrease in lanthanum (La) content in maize plants inoculated with *C. etunicatum*, attributing this effect to the metal binding mechanism employed by AMF. AMF play a pivotal role in mitigating HMs at the fungal mycelium, acting as a physical barrier and through the chelation of HMs by the fungal cell wall chitin. This immobilizes HMs in contaminated soils, curbing their translocation and bioaccumulation within plant tissues. Comparable outcomes have been observed in various crops, such as rice under cadmium (Cd) [31,70], maize under lead (Pb), zinc (Zn), and Cd [71], sorghum under molybdenum (Mo) [72], maize under La [69], and wheat under nickel (Ni) [73] contaminations. The primary mechanism orchestrated by AMF involves the immobilization of HMs using two main strategies—chelation and sequestration—thus alleviating this stress in cereal crops [73]. Furthermore, mycorrhizal plants have been found to produce metal-binding proteins, including metallothioneins, by inducing the expression of related genes [73]. The process of AMF-assisted phytoextraction is facilitated by the production of chelating agents by AMF, metal transformation, and increased bioavailability [73].

AMF play a pivotal role in alleviating abiotic stress in cereal crops. Through diverse mechanisms, such as enhancing the photosynthetic machinery, facilitating osmotic adjustment, and mitigating HMs stress, AMF significantly contribute to bolstering the resilience of cereal plants in challenging environments. The multifaceted benefits of AMF symbiosis underscore their potential for mitigating the detrimental effects of abiotic stressors, ultimately leading to improved yield and quality in cereal crops. Table 1 provides a comprehensive summary of the various AMF species evaluated for their roles in alleviating abiotic stress in cereal crops.

#### 2.1.2. Cereal Mycorrhizal Responses to Biotic Stresses

When considering the induction of resistance to biotic stress by AMF, Table 2 provides a comprehensive summary of the various AMF species employed against biotic stress agents in cereal crops. Numerous studies have demonstrated the role of AMF in managing biotic stress in wheat, including resistance to pathogenic fungi [82,83], bacteria [84,85], and insects [86,87]. Spagnoletti et al. [88] reported that the inoculation with *R. intraradices* improved growth traits and defense mechanisms, and reduced lipid peroxidation and *Fusarium* crown and root rot in winter wheat. AMF compete against pathogenic fungi for colonization sites, photosynthesis, and root space, inducing anatomical changes in root system morphology, rapid detoxification of H_2_O_2_ to mitigate disease severity, and reinforcing the antioxidant defense machinery [88]. Similar trends were noted by Campo et al. [89] in rice inoculated with *F. mosseae* or *R. ntraradices* against *Magnaporthe oryzae*. Prasetyo et al. [90] observed improved tolerance to downy mildew caused by *Peronosclerospora* spp. in maize inoculated with *Enthropospora* sp., *Gigaspora* sp., or *Glomus* sp.

Additionally, AMF application has been frequently associated with improvement in cereal crops’ tolerance to stressful biotic environments by enhancing the plants’ nutritional profile and inducing alterations in epidermal root cells, such as cell wall thickening, and the overaccumulation of secondary metabolites, namely phenolic compounds [88]. Similar mechanisms were observed in mycorrhizal cereals affected by bacterial pathogens, particularly in wheat inoculated with *F. mosseae* against bacterial leaf streak caused by *Xanthomonas translucens* [84,85]. Bhavanam and Stout [92] demonstrated that *R. intraradices* enhanced the oxidative defense machinery against fall armyworm (*Spodoptera frugiperda*) infestation in rice. Charters et al. [93] reported improved root growth and enhanced P and N uptake in *R. irregularis*-inoculated wheat against grain aphids (*Rhopalosiphum padi*). It is widely assumed that AMF can mitigate pathogenic insect infection by modifying root exudates, in addition to leading to the systemic activation of antioxidant enzymes, which significantly reinforces the resilience of cereals to pathogenic insects [93].

### 2.2. Oilseed Mycorrhizal Responses to Stressed Environments

#### 2.2.1. Oilseed Mycorrhizal Responses to Abiotic Stresses

Oilseed crops are frequently exposed to abiotic stresses, which can significantly impact their nutritional value and yield. AMF play a key role in providing protective and beneficial effects to stressed oilseed crops. Table 3 presents the mitigative roles of AMF in oilseeds subjected to drought, salinity, and HMs stresses. Severe water limitation adversely affects the water status and gas exchange of oilseed crops, leading to alterations in photosynthesis and reduced crop yield. However, AMF have been shown to enhance oilseed biomass, water and mineral uptake, photosynthetic apparatus, osmoregulators, secondary metabolism, and defense machinery [94,95,96,97,98,99,100,101]. These beneficial mechanisms have been elucidated in oilseed crops such as soybean [94,95,96,97], sesame [98,99], and linseed [100,101]. For instance, Oliveira et al. [102] found that the inoculation with *R. clarus* improved WUE, physiological traits (leaf water potential (Ψ) and maximum quantum yield of photosystem II (F_v_/F_m_)), and nutrient acquisition in soybean. Similarly, Ghasemi et al. [103] demonstrated enhanced oilseed yield and nutrient content in sesame inoculated with *F. mosseae*.

Salinity negatively impacts the growth stages of oilseed crops, from delaying flowering onset to reducing reproductive structures’ quantum, ultimately affecting the yield and seed oil quality. However, studies have indicated that AMF enhance salinity tolerance in oilseeds, including sunflower [104], safflower [105], dragon’s head [106], and peanut [107]. The AMF-induced tolerance is associated with improvements in nutrient uptake, antioxidant machinery, and a decrease in MDA and Na^+^ levels. Additionally, AMF have been found to enhance phytohormone production against salinity. Indeed, Hashem et al. [108] demonstrated the amelioration of IAA biosynthesis in soybeans inoculated with *F. mosseae*, *R. intraradices*, or *C. etunicatum*. Under saline conditions, IAA plays pivotal roles in nodule vasculature development, AMF–host plants’ signaling, root system development, and amelioration of nutrient assimilation.

Oilseed crops grown in the presence of excessive HMs experience disturbances in nutrient assimilation, carbohydrate metabolism, stunted biomass, and reduced yield. However, AMF have been shown to detoxify HMs contamination in oilseeds by improving nutrient uptake, physiological traits, and antioxidant machinery, and by decreasing MDA content and root-to-shoot HMs translocation. These mechanisms have been demonstrated in sunflower [109] and soybean [110,111]. For instance, *R. intraradices*-inoculated soybean grown under Cu, Pb, and Zn showed improved P acquisition and grain yield [111]. Furthermore, AMF bind HMs through intra- and extra-radical mycelium at the root cortex, and mycorrhizal structures store HMs in the mycelium, acting as a barrier-like strategy that prevents metal translocation to oilseed shoots and seeds. Păun et al. [109] observed that the sunflower plants inoculated with *R. intraradices* and grown under contaminated soils had lower As, Cr, and Ni contents and improved P content compared to control non-mycorrhizal plants. It is suggested that AMF extra- and intra-radical mycelium aid in binding, adsorbing, complexing, precipitating, and crystallizing HMs against AMF hyphal walls. Additionally, GRSP potentially acts as an HMs chelating agent, relying on its affinity properties. Molina et al. [110] reported a decrease in mycorrhizal soybean root Cd, indicating a possible remediation of Cd by *R. intraradices*.

**Table 3:** AMF mitigate abiotic stress in oilseed crops.

Stress	Plant	AMF Species	AMF Colonization Effects	Ref.
Drought				
	Soybean (*Glycine max*)	*R. irregularis*	- Enhanced TSS, proline content, and MAPK transcripts.	[94]
	Flax (*Linum usitatissimum*)	*F. mosseae*, *R. intraradices*	- Enhanced leaf P content and oil yield.	[100]
	Sesame (*Sesamum indicium*)	*F. mosseae*, *R. intraradices*	- Improved Chl index, and N, P, K, Zn, Fe, and Cu content.	[98]
	Soybean (*Glycine max*)	*R. intraradices*, *R. clarus*, *R. aggregatum*, *S. deserticola*, *F. mosseae*, *O. etunicatum*	- Improved biomass, Chl content, g_s_, leaf water relations, and N, P, K, S, Mn, and Cu content.	[97]
	Flax (*Linum usitatissimum*)	*F. mosseae*, *R. intraradices*	- Improved vesicle diameter, yield, and SOD, APX, and POX activity.	[101]
	Sesame *(Sesamum indicum*)	*F. mosseae*, *R. intraradices*	- Improved TSP, P, Chl, flavonoid contents, and seed/oil yield.	[99]
	Soybean (*Glycine max*)	*P. occulum*, *G. gigantea*, *F. mosseae*, *C. etunicatum*, *R. clarus*	- Higher growth traits (pod number, seed number, and seed DM) and oilseed proline.	[95]
	Soybean (*Glycine max*)	*G. clarum*, *G. mosseae*, *Gigaspora margarita*	- Enhanced yield, and seed CAT and POX activity. - Decreased MDA and proline contents. - Up-regulated *CAT* and *POX* expression and down-regulated proline metabolism genes (*P5CS*, *P5CR*, *PDH*, and *P5CDH*).	[112]
	Sesame (*Sesamum indicum)*	*R. intraradices*, *F. mosseae*	- Improved grain yield, oil content, and N and P content.	[103]
	Soybean (*Glycine max*)	*G. mosseae*	- Improved glucose exudation, and β-glucosidase and acid phosphomonoesterase.	[113]
	Soybean (*Glycine max*)	*R. clarus*	*-* Improved plant height, water potential, WUE, F_v_/F_m_, and N and K content.	[102]
Salinity				
	Sunflower (*Helianthus annuus*)	*R. irregularis*	- Lower Na^+^ and MDA content. - Improved biomass and nutritional profile (K^+^, Mg^2+^, Ca^2+^, N, P), soil enzyme activities (CAT, dehydrogenase, phosphatase, fluorescein diacetate hydrolysis).	[104]
	Safflower (*Carthamus tinctorius*)	*R. intraradices*, *F. moseae*	- Improved shoot and root DM, stem and root heights, proline, pigment, P, N, Mg contents, and antioxidant enzyme activity.	[105]
	Soybean (*Glycine max*)	*F. mosseae*, *R. intraradices*, *C. etunicatum*	- Improved nitrogenase and IAA synthesis, and lower H_2_O_2_ and MDA content.	[108]
	Iberian dragon’head (*Lallemantia iberica*)	*F. mosseae*	*-* Ameliorated seeds’ oil and mucilage composition.	[106]
	Groundnut (*Arachis hypogaea*)	*R. irregularis*, *F. mosseae*	- Reduced MDA content. - Improved A_n_, RWC, plant height, osmolyte production, SOD, POX, CAT, APX, protein, and pod yielding.	[107]
Heavy Metals				
	Sunflower (*Helianthus annuus*)	*G. intraradices*	- Enhanced P, Chl, carotenoid, SOD, and PPO. - Reduced MDA, As, Cr, and Ni translocation.	[109]
	Soybean (*Glycine max*)	*R. intraradices*	- Lowered Cd accumulation in roots. - Promoted P and Fe abundance in roots.	[110]
	Soybean (*Glycine max*)	*F. mosseae*	- Boosted growth, yield, and P assimilation. - Decreased Cu, Pb, and Zn translocation.	[111]
	Soybean (*Glycine Max*)	*R. Intraradices*	- Improved growth, P acquisition, and grain yield under Cu, Pb, and Zn soil pollution. - Reduced translocation of the HMs.	[111]

APX: ascorbate peroxidase; As: arsenic; Ca: calcium; CAT: catalase; Cd: cadmium; Chl: chlorophyll; Cr: chromium; Cu: copper; DM: dry matter; Fe: iron; Fv/Fm: chlorophyll fluorescence; gs: stomatal conductance; HMs: heavy metals; K: potassium; MAPK: mitogen-activated protein kinase; MDA: malonyldialdehyde; Mg: magnesium; Mn: manganese; N: nitrogen; Na: sodium; Ni: nickel; P: phosphorus: Pb: lead; POX: peroxidases; P5CS: pyrroline-5-carboxylate synthetase; P5CR: pyrroline-5-carboxylate reductase; PDH: pyruvate dehydrogenase; P5CDH: Δ1-pyrroline-5-carboxylate dehydrogenase; PPO: polyphenol oxidase; RWC: relative water content; S: sulphur; SOD: superoxide dismutase; TSP: total soluble proteins; TSS: total soluble sugars; WUE: water use efficiency.

#### 2.2.2. Oilseed Mycorrhizal Responses to Biotic Stresses

AMF have been shown to enhance the resilience of oilseed crops to biotic stress by depriving the plants of nutrients and affecting their vigor [114,115]. Table 4 provides a summary of AMF application and their alleviative role in oilseeds under biotic stress. Recent research has demonstrated that AMF application can ameliorate growth traits, enhance nutrient uptake, and mitigate the disease severity in soybean caused by pathogenic fungi [114,116], nematodes [117], and insects [118]. For example, Spagnoletti et al. [119] concluded that soybean inoculated with *R. intraradices* reduced the severity of charcoal rot caused by *Macrophomina phaseolina*. AMF compete with pathogenic fungi for colonization sites, space, and photosynthesis. Similarly, Bán et al. [120] showed that the inoculation with AMF belonging to the *Glomus* genus led to localized and systemic resilience to white rot (*Sclerotinia sclerotiorum*) in sunflower.

Emerging research has highlighted the potential of AMF to mitigate the impact of nematode and insect infestations in oilseed crops [120]. Investigations into soybean and other oilseed crops have revealed that AMF application can ameliorate the negative effects of nematode infestations, as well as reduce the damage caused by insect pests [120]. These findings suggest that AMF-induced modifications in root physiology and defense responses contribute to enhanced resilience against nematodes and insects, thereby safeguarding the health and productivity of oilseed crops [112,113]. In-depth studies have elucidated the cellular and physiological modifications induced by AMF in oilseed crops in response to biotic stresses. Mechanisms such as alterations in cell wall composition and the accumulation of phenolic and fluorescent compounds have been identified as key contributors to the ability of oilseed crops to withstand pathogenic fungal attacks [117]. These insights into the intricate interplay between AMF symbiosis and the defense responses of oilseed crops provide valuable knowledge for the development of strategies to enhance biotic stress resilience in agricultural settings.

In summary, the collective body of evidence underscores the significant potential of AMF in fortifying oilseed crops against a spectrum of biotic stresses, ranging from pathogenic fungi to nematodes and insects. The elucidation of diverse mechanisms underlying AMF-induced resistance provides a foundation for future research and practical applications aimed at enhancing the sustainability and productivity of oilseed cultivation in the face of biotic stress challenges.

## 3. Molecular Strategies Contributing to Cereal and Oilseed Tolerance to Environmental Stresses

### 3.1. Molecular Mechanisms behind Cereal Mycorrhiza Responses

#### 3.1.1. Molecular Mechanisms behind Cereal Mycorrhiza Responses to Abiotic Stresses

**Figure 1:**
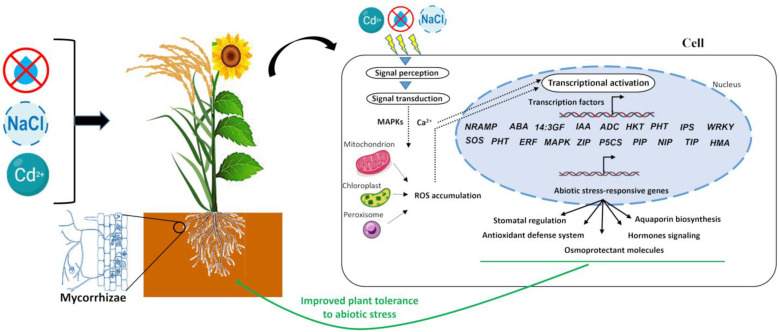
Molecular mechanisms behind cereal/oilseed–AMF interaction responses to abiotic stresses. After their perception, abiotic stress signals are transduced in the cytoplasm of the mycorrhizal plant cell where they induce the production of ROS by many organelles such as mitochondrion, chloroplast, and peroxisome [48,49]. The accumulation of ROS triggers the activation of various transcription factors that regulate the expression of abiotic stress-responsive genes, thereby stimulating many functioning pathways including stomatal regulation, aquaporin biosynthesis, antioxidant defense system, hormone signaling, and osmoprotectant molecules, leading the improved plant tolerance to abiotic stress [122]. ABA: abscissic acid; ADC: arginine decarboxylase; ERF: ethylene-responsive factors; HMA: heavy metal ATPase; HKT: high-affinity K^+^ transporter; IAA: indole-3-acetic acid; IPS: D-myo-inositol-3-phosphate synthase; 14-3GF: 14-3-3-like protein GF14; MAPK: mitogen-activated protein kinase; NIP: nodulin 26-like intrinsic protein; NRAMP: natural resistance-associated macrophage protein; P5CS: pyrroline-5-carboxylate synthetas; PHT: phosphate transporter; PIP: plasma membrane intrinsic protein; SOS: overly sensitive; TIP: tonoplast intrinsic protein; ZIP: zrt/irt-like protein.

Cereals respond to abiotic stress by recruiting avoidance and/or tolerance mechanisms that encompass morphological, physiological, and molecular responses. Numerous mechanisms enhancing cereal tolerance to abiotic stress by AMF have been identified, involving a variety of molecular responses at different plant levels [122] (Figure 1).

The molecular mechanism of AMF on water acquisition and transport in plants under abiotic stress strongly suggests its impact on aquaporin (AQP) that mediates water transport in plants [123,124,125]. In *Zea mays*, plants inoculated with *R. irregularis* showed an increase in the expression of the AQP genes *ZmPIP1;1*, *ZmPIP1;2*, and *ZmPIP2;1* under drought stress [126]. Similarly, Quiroga et al. [122] reported that the expression of the AQP genes *ZmPIP2*;*2* and *ZmPIP2*;*6* in the roots of maize plants inoculated with AMF was up-regulated under drought stress. Asadollahi et al. [127] found that the AQP gene expression of the NIP subfamily (*TaNIP1-10*, *TaNIP3-3*, *TaNIP3-4*, *TaNIP1-5*, and *TaNIP1-6*) and PIP subfamily (*TaPIP2-7*) transporters was up-regulated in wheat plants inoculated with AMF under water stress conditions. Overexpression of the *TaNIP1-10*, *TaNIP3-3*, and *TaNIP3-4* genes was mainly up-regulated by mycorrhizal inoculation.

Nutrient exchange between plants and AMF is the main benefit for both symbiotic partners [128,129]. Most glycophytes tolerate salinity by limiting the uptake of Na^+^ and Cl^−^ while maintaining the uptake of macronutrients such as K^+^ that enable cereal plants to have salinity tolerance [130]. AMF-colonized maize plants increased the expression of the *ZmAKT2* and *ZmSKOR* genes under salt stress to maintain a high K^+^/Na^+^ ratio, mainly due to K^+^ retention rather than Na^+^ exclusion [130,131]. AMF were also found to contribute to the regulation of ion homeostasis-related genes (*OsSOS1*, *OsNHX3*, *OsHKT1;5*, and *OsHKT2;1*), thereby improving salt tolerance in rice plants by decreasing Na^+^ distribution between roots and shoots [63,132]. Phosphate transporter (PT) proteins enhance inorganic orthophosphate (P_i_) uptake in mycorrhizal plants under abiotic stress conditions [133,134]. The Pi transporter can be strongly induced by AMF [133]. The P_i_ transport is carried out by P_i_/H^+^ symporters from the *Pht1* gene family [135,136,137]. The expression of these mycorrhizal proteins enabled P_i_ to be transferred in the form of polyphosphates and then absorbed via plant PTs, composed of four subfamilies, PT1-4 [138]. It has been shown that *PT*: *ZEAma: Pt1;6* expression is uniquely induced in maize plants with mycorrhiza formation and plays a crucial role in P_i_ acquisition in P-deficient soil [133]. Sorghum plants inoculated with *R. irregularis* showed an increase in *SbPT5* expression, which plays a key role in improving P_i_ uptake, linked with an increase in cereal plants’ tolerance under abiotic stresses [123,136,139].

In plants subjected to severe metal stress, a notable increase in P_i_ acquisition has been observed in response to inoculation with AMF [140]. This reduction in HMs toxicity is likely attributed to the heightened exchange of nutrients facilitated by AMF and the host plants. This nutrient increase has been strongly associated with the up-regulation of three specific genes playing key roles in the absorption of Cd into root tissues—*Zm00001d014669*, *Zm00001d017292*, and *Zm00001d051936*—in AMF-inoculated maize [141]. Furthermore, the expression of *OsCAL1* and *OsHAM2* genes is implicated in the absorption of toxic metal elements in the root tissues [141]. Notably, the mitigation of HMs’ detrimental effects in cereal plant root tissues may also be ascribed to the mRNA expression of various antioxidant enzymes, which induce improved tolerance in host plants [142]. Research has indicated that genes encoding glutathione synthetase, specifically *Zm00001d007670* and *Zm00001d040146*, were up-regulated in stressed plants vs. controls, suggesting that AMF-inoculated maize further enhances mRNA expression to mitigate the effects of metal stress-induced ROS [141]. AM-inoculated cereals may trigger the antioxidant synthesis-related genes, thereby bolstering tolerance to HMs stress.

To gain a deeper understanding of the mechanisms underlying water deficit stress, a proteomic analysis on mycorrhizal *Sorghum bicolor* L. plants identified 51 differentially accumulated proteins in response to water deficit stress. Notably, different metabolic pathways in AMF–sorghum leaves involved proteins related to energy (ATP synthase β, ATP synthase-24kDa) and carbon (sucrose-phosphatase, malate dehydrogenase, triosephosphate isomerase), oxidative phosphorylation (mitochondrial-processing peptidase), and sulfur (thiosulfate/3-mercaptopyruvate sulfurtransferase) metabolisms [143]. In a related study, wheat plants inoculated with AMF exhibited the down-regulation of the expression of sugar efflux transporter genes and invertases (beta-fructofuranosidase) involved in the conversion of sucrose to fructose and glucose. Additionally, the expression of trehalose-6-P synthase genes and two trehalose-6-P phosphatases responsible for trehalose biosynthesis was up-regulated in wheat plants under salt stress conditions, indicating that osmoregulation and the accumulation of osmolytes such as sugars, amino acids, and trehalose were modulated by AMF, thereby enhancing plant tolerance to salt stress [144]. Under saline stress conditions, the accumulated availability of polyamines in plants further inoculated with AMF may induce a molecular mechanism to improve plant adaptation to saline soils. The up-regulation of relative genes *ADC*, *ODC*, *SAMDC*, *SPDS2*, and *PAO* in plants inoculated with AMF and subjected to salinity stress suggests the potential for enhanced osmoregulation and the optimization of photosynthesis under stress conditions, leading to changes in the genes linked to these processes [145]. It has been observed that mycorrhizal plants recorded the increased expression of *TRINITY_DN122268_c7_g5* genes (PsbP family protein of PS II reaction center) under alkaline stress conditions. This up-regulation of genes enabled oxygen release to promote photosynthesis and reduced ROS accumulation in cells by down-regulating NADH-ubiquinone oxidoreductase-related genes in the mitochondrial electron transport chain under stress [146].

Additionally, AMF have been found to regulate numerous genes related to PS II, PS I, and the chlorophyll protein complex in maize under low-temperature stress conditions [146]. The symbiotic association between wheat and AMF (*F. mosseae*) not only affects the transcription profile of plant growth but also the cell wall and membrane components. The genes associated with symbiotic plants under drought were the most differently expressed among the 114,428 genes expressed in wheat roots. The genes associated with the metabolic processes of carbohydrates, lipids, cellulose synthase activity, membrane transports, N-compounds, and chitinase activity were found to be the most differentially expressed. The biosynthesis of phytohormones such as ABA in stressed plants helps to improve drought tolerance, while in the meantime, it increases and establishes the AM symbiosis [147]. ABA accumulation in stressed plants induces the expression of genes encoding *D-myo-inositol-3-phosphate synthase* (*IPS*) and *14-3-3-like protein GF14* (*14-3GF*), responsible for ABA signal transduction, has been shown to be involved in the activation of 14-3-3 protein and aquaporins (*GintAQPF1* and *GintAQPF2*) in AMF. Consequently, the expression of *IPS* and *14-3GF* is responsible for AMF actions in improving the drought tolerance of maize plants [74]. These findings underscore the impact of AMF on cereal responses to stresses, shedding light on the intricate molecular mechanisms underlying plant–fungal interactions in challenging conditions.

#### 3.1.2. Molecular Mechanisms behind Cereal Mycorrhiza Responses to Biotic Stresses

**Figure 2:**
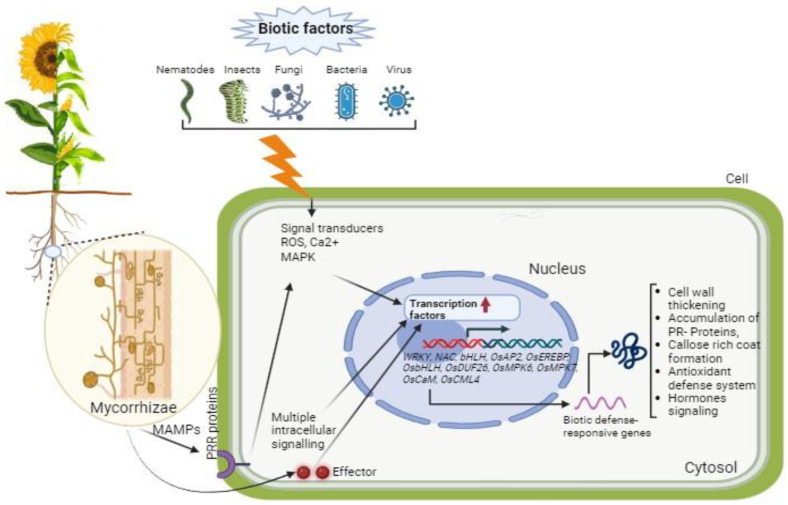
Molecular mechanisms of cereal/oilseed–arbuscular mycorrhizal fungi (AMF) responses to biotic stresses. The diagram depicts the intricate molecular mechanisms involved in the response of cereals/oilseeds to biotic stresses mediated by AMF. Reactive oxygen species (ROS) and Ca^2+^ act as crucial transducers, while mitogen-activated protein kinase (MAPK) cascades play a central role in the crosstalk between Ca^2+^ and ROS, contributing to signal production following exposure to biotic stress [94,149]. Pathogenic microorganisms and plant defense activators, including AMF, bear microbe-associated molecular patterns (MAMPs) on their surface, along with effector proteins that are secreted externally or internally to plant cells. Recognition of MAMPs and effector molecules by pattern recognition receptors (PRRs) leads to the activation of MAMP-triggered immunity (MTI) or effector-triggered immunity (ETI), respectively [150,151]. The activation of defense responses can result in a hypersensitive response (HR), characterized by rapid, localized necrosis at the pathogen’s point of entry. Salicylic acid (SA) and jasmonate/ethylene (JA/ET) orchestrate the plant’s response to biotic stress, with hormones, secondary metabolites, priming agents, and other cytoplasmic chemicals ultimately up-regulating transcription factors (TFs), pathogenesis-related genes (PRs), heat shock protein (HSP) genes, and other defense-related genes [152]. These molecular events collectively contribute to the plant’s protection against biotic stresses.

The protective effects of AMF against biotic stresses are believed to be mediated by underlying molecular pathways and the regulation of gene expression [148] (Figure 2).

For instance, the resistance abilities of wheat to certain biotic stresses could be enhanced by AM fungal colonization, partly explained by the up-regulation of genes belonging to the *WRKY*, *NAC*, and *bHLH* TF families [153]. In a study involving *R. intraradices*-inoculated rice infected with *Magnaporthe oryzae*, different functional categories of genes were up-regulated in the leaves, including those involved in transcriptional control, signal transduction, and protein synthesis, as well as defense and stress responses [154]. The study revealed the up-regulation of TFs such as *OsAP2*, *OsEREBP* (members of the *AP2/EREBP* family), and *OsbHLH* (basic helix–loop–helix), along with genes implicated in signal transduction processes, namely *OsDUF26* (*domain unknown function 26*) and *OsMPK6* (*mitogen-activated protein kinase 6*). Additionally, a significant increase in the expression of genes playing a regulatory role in the plant defense response, including *OsMPK7*, was observed [154]. The study highlighted AM-induced systemic alterations in the expression of genes involved in Ca^2+^-mediated signaling processes. The expression of *OsCaM* (*calmodulin*) and *OsCML4* (*calmodulin-like 4*) genes was markedly activated in mycorrhizal rice in response to infection, indicating a role for Ca^2+^-mediated signaling processes in systemic resistance to AM-induced pathogen infection in rice plants [154].

Furthermore, *Trichoderma asperellum* has been identified as a potential biocontrol agent capable of suppressing rice blast caused by *M. oryzae* [155]. Inoculation with *T. asperellum* enhanced the expression of defense-related genes, which occur during systemic resistance, such as *LOX-RLL* and *PR1b* in rice plants [155]. In the leaves of *R. intraradices*-inoculated cultivated rice infected with *M. oryzae*, the up-regulation of genes encoding endo-1,3-ß-glucosidases (*Os07g0539400* and *Os07g0600700*) and cellulose synthases (*Os08g0345500*, *Os08g0160500*, and *Os09g0428000*) was observed compared with uninoculated cultivated rice infected with *M. oryzae* [91]. In another study, the expression levels of defense-related genes such as *peroxidase* (*POX*), *chitinase* (*CHI1*), and *nonexpresser of pathogenesis-related 1* (*NPR1*) were monitored in mycorrhizal and non-mycorrhizal wheat plants infected by *Blumeria graminis* f. sp. tritici, which causes powdery white sporulating colonies to appear on wheat leaves and stems [83]. Surprisingly, no up-regulation in the expression of the targeted genes (*POX* and *CHI1*) was detected in the leaves of wheat plants inoculated by *F. mosseae* challenged with *B. graminis* 21 h after infection. In contrast, *POX*, *CHI1*, and *NPR1* were up-regulated in wheat leaves in response to root colonization by *F. mosseae*, in the absence of *B. graminis* infection.

Signaling molecules like salicylic acid (SA), jasmonic acid (JA), and ethylene (ET) are pivotal regulators of defense mechanisms against pathogens and play crucial roles in coordinating systemic resistance responses [152]. They regulate gene expression and activate defense pathways. SA is recognized for activating responses against biotrophic pathogens, while plant responses to necrotrophic pathogens are predominatly governed by the JA and ET pathways [152]. In a study conducted by El-Sharkawy et al. [156], molecular investigation revealed that mycorrhizal colonization with an AMF inoculum containing three types of AMF (*R. irregularis*, *R. clarum*, and *G. gigantean*) up-regulated the defensive gene *ethylene response factor protein 3* (*JERF3*) in wheat plants infected by *Puccinia striiformis* f. sp, the causal agent of stripe rust. Furthermore, two SA-related genes encoding carboxyl methyltransferases, *Os11g0260100* and *Os01g0701700*, were up-regulated in the leaves of cultivated rice infected by *M. oryzae* after inoculation with *R. intraradices* [91].

Secondary metabolites, including polyphenols, particularly flavonoids, and chlorogenic acid are increased during systemic resistance and play crucial roles in enhancing defense responses to various invading pathogens [157]. In a study by Rashad et al. [158], genes that regulate the three main parts of the polyphenol biosynthesis pathways—phenylpropanoid, flavonoid, and chlorogenic acid—in wheat leaves infected with *P. striiformis* f. sp. tritici in response to mycorrhizal colonization (*R. clarum*, *G. gigantean*, and *R. irregularis*) were investigated. Mycorrhizal colonization of the 7-day infected wheat plants led to a significant up-regulation of seven genes: *CHI2 (chalcone isomerase)*, *F3H (flavanone 3-hydroxylase)*, *F3′H (flavonoid 3′ hydroxylase)*, *DFR (dihydroflavonol-4-reductase)*, *FLS1 (flavonol synthase 1)*, *AN2 (anthocyanin 2)*, and *HCT (hydroxycinnamoyl-CoA shikimate hydroxycinnamoyl transferase)*. However, *PAL1 (phenylalanine ammonia lyase 1)*, *AN 1 (anthocyanin 1)*, and *C3H (4-coumarate 3-hydroxylase)* were significantly down-regulated. In the leaves of rice plants inoculated with *R. intraradices* and infected with *M. oryzae*, no shikimate pathway genes with altered expression were detected, while only down-regulated phenol-related genes (*Os02g0749700* and *Os12g0258700*) were observed [91].

### 3.2. Molecular Mechanisms behind Oilseed Mycorrhiza Responses to Stressed Environments

#### 3.2.1. Molecular Mechanisms behind Oilseed Mycorrhiza Responses to Abiotic Stresses

Oilseed crops rely on a myriad of molecular mechanisms to cope with abiotic stresses. The colonization of oilseed crops by AMF instigates multiple molecular changes that play a pivotal role in the plants’ responses to these stresses [112,159]. Mitogen-activated protein kinase (MAPK) cascades have been identified as crucial components in the plants’ defense against abiotic stresses. The establishment of a symbiotic relationship between oilseed plants and AMF has been shown to bolster their tolerance to abiotic stress, with this enhancement attributed to the activation of fungal MAPK responses and the intricate interplay between fungal and plant MAPK cascades [94,149]. Under drought conditions, *R. irregularis* MAPKs, which exhibited high similarity with soybean MAPKs, displayed distinct patterns of gene expression. In mycorrhizal soybean roots, drought stress increased the levels of fungal *MAPKs* (*RiMAPK1*, *RiMAPK3*, and *RiMAPK4*) and soybean *MAPKs* (*GmMAPK3-2*, and *GmMAPK5*) transcripts [94]. Examination of *R. irregulare*-inoculated sunflower revealed differential expression of genes (DEGs) associated with stress response, notably including chitinase and germin-like proteins, along with genes encoding polypeptides exhibiting protease activity (carboxypeptidases). Among the identified DEGs were those encoding an nrt1 protein of the ptr family, a glutathione-transferase, two UDP-glycosyltransferases, an inorganic phosphate transporter, and a lysM domain receptor-like kinase 3 [160].

Root transcriptomes of droughted common bean plants (cv. BAT 477) treated with three AMF species (*Acaulospora scrobiculata*, *G. rosea*, and *G. clarum*) revealed 1589 transcripts uniquely controlled by AMF inoculation. Significant changes in gene expression were observed in roots treated with AMF under water shortage. Exclusive genes to arbuscule-containing cells, *Glucan 1*,*3 β-Glucosidase* and *aquaporin PvPIP2;3,* were identified [159]. Aquaporins (e.g., *PvPIP2;3*) offer a low-resistance pathway for water, providing plant tissues with more control over water movement through membranes [161]. *PIP* and *TIP* isoforms—key players in water transport—suggest a significant alteration in host plant AQP activity due to mycorrhizal symbiosis [159,162]. In common bean leaf samples subjected to water deficit, AMF-inoculated plants displayed reduced expression levels of *PvPIP1;2*, while three other genes, *PvTIP1;1*, *PvPIP1;1*, and *PvPIP2;1,* exhibited heightened expression levels. Additionally, four genes, *PvPIP1;3*, *PvPIP2;5*, *PvPIP2;3*, and *PvPIP2;6,* were found to be up-regulated in AMF-inoculated plants [159]. These findings indicate that the increased biosynthesis of proteins and lipids may play a role in safeguarding soybean metabolism, promoting growth, and supporting development under drought stress. The study provides evidence that the up-regulation of *Glycine max-Sucrose Synthase* (*GmSuSy*) may contribute to triggering alterations at the transcriptional level. Additionally, catalase and peroxidase genes were found to be overexpressed in soybeans inoculated with AMF under drought [112].

Under abiotic stresses such as iron (Fe) deficiency, the representative gene associated with ferric reductase activity (*HaFRO1*) was observed to be activated following AMF–root interactions in sunflower plants. In the same conditions, the presence of AMF (*R. intraradices*, *G. mosseae*, *G. aggregatum*, and *G. etunicatum*) was linked to a significant increase in sunflower biomass, accompanied by an up-regulation of Fe and Zn transporters (*HaIRT1*, *HaNramp1*, and *HaZIP1*) [163]. Inoculation of soybean plants with *G. clarum*, *G. mosseae*, and *G. margarita* was found to enhance N fixation under drought [112] through nodulation and positive regulation of genes encoding NO_3_^−^ transporters such as *NRT1* under mineral nutrient stresses [164]. The study also observed the strong impacts of salinity on the expression of nitrate transporter (*NRT2.4*) and phosphate transporter (*PHO1*) genes in plants treated with *R. irregularis*. Under salinity conditions, *R. irregularis*-inoculated pistachios—exploited for their kernel oil—exhibited a marked increase in the expression of the ion transporter *SOS1*, *SKOR*, and *CCX2* genes responsible for Na^+^/K^+^ ratio, K^+^, and Ca^2+^ regulation [165]. Notably, the study revealed that the *PIP2.4* gene was overexpressed in the roots of both AMF and non-AMF plants under salinity conditions.

The interaction between oilseeds such as soybean and AMF induces notable alterations in root metabolism, presenting an opportunity for metabolomics studies to elucidate the mechanisms underlying AMF–plant symbioses. A promising approach to harness soil AMF resources for improved soybean cultivars capable of sustained colonization and enhanced responsiveness involves the selection of cultivars based on their genetic variability [166]. Metabolomics analysis on drought-tolerant and drought-susceptible sesame genotypes to unravel their adaptation mechanisms in response to water scarcity identified ABA, amino acids (tryptophan, phenylalanine, valine, leucine, tyrosine, saccharopine, and 2-aminoadipate), Gamma-aminobutyric acid (GABA), and organic acids (glutaric acid and 2-methylcitric acid) as the most significantly accumulated metabolites under drought stress, shedding light on the sesame’s response to water scarcity [167].

There exist noteworthy parallels in phytohormone responses, particularly IAA signaling, between rhizobial and beneficial fungal symbioses. The symbiotic efficacy of oilseed plants with *Bradyrhizobium*, another microsymbiont, is significantly bolstered by the presence of the mutualistic fungus *Phomopsis liquidambari* [168]. In the context of peanuts (used for their kernel oil), a comprehensive examination of the transcriptional response of key nodulation signaling genes, including *SymRK*, *CCaMK*, and early nodulin genes *NIN* and *ENOD40*, provides insights into the involvement of IAA signaling in *P. liquidambari*-mediated nodulation signaling activation [6]. The modulation of IAA signaling within ternary *P. liquidambari*–peanut–*bradyrhizobial* interactions revealed that *P. liquidambari* induces both local and systemic IAA signaling, as evidenced by the expression of the IAA-responsive reporter, *DR5:GUS*. Moreover, ethylene-responsive factors (*ERFs*) play a critical role in the fitness and adaptation of oilseed plants to abiotic stress conditions. Through phylogenetic analysis, 160 soybean *ERF* genes were identified, distributed across 20 chromosomes, and classified into eight groups. Notably, the Group VII *ERF* gene *GmERF75*, exhibiting strong ABA responsiveness, was comprehensively characterized. Overexpression of *GmERF75* in soybean hairy roots was observed to significantly enhance root development in response to exogenous ABA and salt stress, demonstrating its potential role in stress adaptation [168,169].

#### 3.2.2. Molecular Mechanisms behind Oilseed Mycorrhiza Responses to Biotic Stresses

Several plausible mechanisms have been proposed to underline mycorrhiza-induced protection in oilseed crops, encompassing improvements in plant nutrient assimilation, alterations in the rhizosphere, and the induction of host defenses. Spagnoletti et al. [170] reported an increased resistance to charcoal rot, which has been observed in both P-fertilized soil and mycorrhizal soybean plants, thereby enhancing the potential role of AMF in conferring bioprotection to the host through improvements in plant vigor and nutrient status. However, the molecular mechanisms that monitor and regulate this process are still not fully understood, and the available data are primarily related to nutrient transporter genes on both the AMF (*GmosPT*, *GiPT*, and *GigmPT* in *F. mosseae*, *R.intraradices*, and *G. margarita*, respectively) and oilseed crops (*GmPHT1;6*, *GmPHT1;7*, *GmPHT1;10*, *GmPHT1;12*, and *GmPHT1;13*) during symbiosis establishment (reviewed by Slimani et al. [171] and references therein). AMF can also directly manage pathogens through intricate interactions in the rhizosphere. Previous studies have reported the AMF impact on sunflower [172] and sesame plants’ enemies [173]. Spagnoletti et al. [114] demonstrated that disease reduction in AMF-inoculated soybean plants may be attributed to the competition between the two fungi (*M. phaseolina* vs. *R. intraradices*) for common resources such as space, infection sites, and photosynthetic products. Competition for space is a common mechanism to control diseases in various AMF–belowground interactions. Another mechanism may be attributed to the root cell lignification prompted by AMF inoculation, which underlies the multifaceted interactions of AMF within the mycorrhizosphere environment and the roots to cope with their host diseases [172]. To create an improved rhizosphere environment, AMF can act either alone [114,174] or synergically in consortium with other AMF [175,176] and/or in combination with other beneficial microflora [172,173,177] to address causal agents of oilseed crops’ diseases. In soybean plants inoculated with *Cylindrocladium parasiticum*, AMF demonstrated a heightened presence of phenolic acids compared to uninoculated plants, suggesting that root exudates may constitute a multifaceted protective mechanism employed by AMF against red crown soybean rot [177]. Furthermore, qRT-PCR results have indicated a more robust induction of transcripts for the tested pathogen defense-related (PR) genes (*PR2*, *PR3*, *PR4*, *PR10*) when a combined inoculation (AMF–Rhizobium) was applied to infested soybean roots.

Root exudates continue to play a pivotal role in plant disease management during plant–AMF interactions. However, the molecular mechanisms of these components and their relevance to oilseed plants remain complex and highly case-specific, highlighting the need for further research in this area. Strigolactones (SLs) are recognized as multifunctional compounds present in root exudates, serving as key signaling molecules not only for AMF but also for other organisms within the surrounding root system. They play a crucial role in regulating various biotic interactions [178,179]. Notably, SLs may contribute to host bioprotection by selectively recruiting certain microbes, particularly those that antagonize root pathogens [180]. The overexpression of SL genes involved in biosynthesis (*MAX1d*) and signaling perception (*D14* and *MAX2a*) in soybean has been found to significantly modulate the rhizosphere bacterial community, although not the fungal community [181]. Once synthesized and exported into the rhizosphere, SL recognition has been shown to effectively impede pathogen growth, as evidenced in in vitro studies involving SL analogues and certain pathogens, including those targeting oilseed crops [182,183,184]. However, the perception mechanism in non-AM fungi and the downstream signaling components remain largely unknown. Recently, Fiorilli et al. [185] identified *CpD14* as a novel SL receptor in the pathogenic fungus *Cryphonectria parasitica*, demonstrating its ability to efficiently mediate fungal growth inhibition in response to SLs under controlled conditions. In a recent assessment of the effect of *F. mossae* on the fungal community isolated from the roots and rhizosphere of two soybean cultivars, the relative abundance of the two most dominant pathogenic fungi, *F. oxysporum* and *Rhizoctonia solani*, decreased in the roots and rhizosphere soils upon AMF inoculation [186]. The impact of *F. mossae* inoculation on the root and rhizosphere community was found to be dependent on the soybean cultivar and cropping regime. Interestingly, two crop cultivars, exhibiting differential affinity towards the same AMF strain, may not respond similarly against the same pathogenic fungus. Among the two soybean cultivars used, the resistant cultivar demonstrated a prompt and enhanced resistance towards root rot pathogens after successful mycorrhization by *F. mossae* inoculation [187].

Host defense induction is a pivotal outcome of plant–AMF interactions, particularly in the context of various oilseed crops. Numerous studies have underscored the enhanced resistance of AMF-colonized plants to pathogens, as well as the systemic protection observed in non-inoculated root fragments and aerial plant organs in response to belowground attackers. These findings collectively emphasize the involvement of plant defense mechanisms triggered by AMF [174,188]. Plant innate immunity plays a crucial role in distinguishing between ‘friendly’ and pathogenic organisms by recognizing structurally conserved microbe-associated molecules, commonly referred to as microbe-associated molecular patterns (MAMPs or PAMPs in the case of pathogens) [150,151]. The perception of MAMPs typically occurs through plant cell surface-anchored pattern recognition receptors (PRRs), leading to the induction of responses such as MAMP-triggered immunity (MTI), PAMP-triggered immunity (PTI), and DAMP-triggered immunity, collectively referred to as pattern-triggered immunity (PTI) [189,190]. MTI remains a crucial immune response in plants, with research dedicated to deciphering the recognition mechanism of MAMPs by PRRs and the complex network underlying signaling events [191,192], as well as the downstream responses. However, plant resistance can be compromised when pathogens overcome the host plant’s defenses, leading to effector-triggered susceptibility and the successful reproduction of the attacker, often resulting in disease symptoms [193]. Pathogens have evolved mechanisms to suppress PTI, leading to the effector-triggered susceptibility of the plant to the disease [149].

Mycorrhiza-induced resistance (MIR) represents an enhanced defensive strategy in response to colonization by AMF [194]. Given that AMF reside within the plant, the colonization process resembles infection by a biotrophic pathogen, with the initial defense responses commonly induced by these two types of microorganisms [195]. Notably, AMF can suppress plant immunity by secreting specific effectors and subsequently establishing a successful infection [196]. AMF exerts immune suppression through calcium/calmodulin kinase DMI3, which represses early-acting defense genes [197]. The examples hereafter of MIR in oilseed crops and the molecular mechanisms underlying the involved defense responses underscore the intricate interplay between plants and AMF in the context of host defense induction. Studies have shed light on the strong induction of plant defense mechanisms in response to AMF in infected soybean plants, enabling them to combat soilborne pathogens such as *Macrophomina phaseolina* and *F. virguliforme* [115,121,181]. The up-regulation of *PAL* and *Chalcone synthase* (*CHS*) genes in mycorrhized *M. phaseolina* plants has been observed, indicating a robust induction of plant defense mechanisms [121]. Proteomic analysis studies conducted by Bai et al. [198] have confirmed the dual role of *F. mossae* in inducing certain defense-related proteins (glucan 1,3-beta-glucosidase, chalcone isomerase, calcium-dependent phospholipid binding proteins) and providing the required energy for the main soybean metabolic processes. Similarly, in sunflower plants, several genes belonging to the PPP have exhibited targeted responses to combat *Rhizoctonia* root rot [174]. PAL activity has been identified as a reliable indicator of plant resistance expression, preceding other defense responses such as cell wall lignification and the accumulation of chlorogenic acid, flavonoids, and anthocyanins in sunflower plants. The up-regulation of the *PAL* gene also triggers systemic defense responses through the SA pathway, leading to the expression of *PR1* defense genes in both mycorrhizal and non-mycorrhizal soybean roots infected by *M. phaseolina* [117]. The mycorrhizal hosts exhibit a faster and stronger defense response, indicating a ’primed state’ as a mechanism of mycorrhiza-induced resistance or tolerance in soybean [149,188].

The intricate molecular mechanisms underlying the induction of plant defense responses in the context of AMF–plant interactions continue to be an enigmatic area of study. However, the integration of newly developed multi-omics approaches has begun to unveil the diversified responses and the differential and overlapped pathways that (oilseed) plants employ to strengthen symbiosis with beneficial organisms and counteract detrimental ones.

## 4. Concluding Remarks

The imperative need to sustainably feed a rapidly expanding global population in the face of climate change underscores the critical importance of understanding the impact of AMF on the performance of cereal and oilseed crops under diverse environmental stresses. This understanding is pivotal for promoting climate-resilient sustainable agriculture. Our review has synthesized the research on the benefits of mycorrhizal symbiosis for oilseeds and cereals, elucidating how these crops modulate their behavior and defense mechanisms under biotic and abiotic stresses when inoculated with AMF. However, our incomplete understanding of the fundamental regulatory processes driving AMF-treated cereal and oilseed tolerance to (a)biotic stressors limits the effective application of AMF at different scales. This limitation is primarily attributed to the intricate complexity of the underlying mechanisms of this response. Nevertheless, the unveiling of this molecular machinery has commenced through advanced omics techniques. Furthermore, it is crucial to identify the critical pathways orchestrating the enhancing effect of AMF on cereal and oilseed crops under diverse environmental stresses. To fully comprehend the mechanisms and pathways involved in AMF-induced resistance in these crops under a changing climate, future studies should focus on cutting-edge technologies such as high-throughput omics approaches, advanced imaging techniques, functional genomics, epigenomics, and systems biology. Additionally, interdisciplinary collaborations integrating fields like bioinformatics, computational biology, (big) data analytics, and artificial intelligence will be crucial for analyzing and interpreting the vast amount of complex data generated by these studies. This comprehensive approach will provide deeper insights into the intricate molecular processes underlying AMF-mediated resistance in cereal and oil crops and pave the way for developing innovative strategies to enhance their productivity and resilience in the face of climate change.

The insights presented in this review shed light on the transformative potential of mycorrhizal technologies as a paradigm-shifting approach to agricultural practices, with profound implications for future agricultural policies and sustainable practices. The exploration of AMF as a sustainable alternative to mineral fertilizers and pesticides offers a compelling avenue for reducing the environmental footprint of farming practices. By enhancing nutrient absorption and alleviating plant stress, AMF offer a promising solution to the challenges posed by climate change-related stressors on crop productivity. Understanding the molecular mechanisms underlying the influence of AMF on crops opens up opportunities for the development of targeted strategies to mitigate environmental stress. This knowledge can inform the formulation of agricultural policies aimed at promoting the adoption of mycorrhizal technologies to enhance crop resilience and productivity while reducing reliance on conventional chemical inputs. The potential of mycorrhizal technologies extends beyond crop production to encompass benefits for agroecological systems and organic farming. By mitigating soil damage and addressing yield decline exacerbated by changing climate conditions, these technologies represent pioneering solutions for the agricultural challenges of the future. As the demand for sustainable agricultural solutions continues to grow, integrating mycorrhizal technologies into agricultural policies and practices holds promise for enhancing crop productivity, reducing the environmental impact, and ensuring the long-term sustainability of agricultural systems. Moreover, this review paves the way for the targeted development of biofertilizers enriched with stress-tolerant AMF strains, capable of bolstering plant resilience in diverse agroecosystems. Our research also emphasizes the potential for integrating knowledge of AMF compatibility and stress tolerance into crop breeding programs, enabling the selection and breeding of crop varieties with enhanced symbiotic interactions with AMF. These advancements are essential for promoting sustainable agricultural practices and enhancing crop productivity in the face of changing environmental conditions.

Considered as the `agricultural technologies of the future’, AM technologies are designed to cultivate healthy, organic agricultural products while safeguarding the integrity of natural resources for future generations. Despite the historical success of mycorrhizal technologies, widespread adoption by farmers has been hindered by challenges in translating successes observed in greenhouse settings to real-world agricultural fields. The industrial sector faces obstacles related to inconsistent results, monitoring inoculants in the field, knowledge gaps, and the absence of standardized methodologies for large-scale inoculant production and quality control. By addressing these challenges and harnessing the full potential of mycorrhizal technologies, the agricultural landscape can be revolutionized to embrace more sustainable and resilient practices, thereby paving the way for enhanced agricultural productivity and environmental conservation on a global scale.

In conclusion, the findings presented in this review offer a compelling case for the widespread adoption of mycorrhizal technologies as a cornerstone of sustainable agricultural management. Embracing these technologies has the potential to revolutionize agricultural practices, cultivate healthy organic products, and safeguard natural resources for future generations, making a significant contribution to the long-term sustainability of global agriculture.

## Figures and Tables

**Table 1:** AMF alleviate abiotic stress in cereal crops.

Stress	Crops	AMF	AMF Colonization Effects	Ref
Drought				
	Maize (*Zea mays*)	*Rhizoglomus intraradices*	- Improved growth traits, P, fresh leaf moisture content, WUE, and reduced C/P and N/P.	[30]
	Maize (*Zea mays*)	*R. intraradices*	- Amelioration of maize growth, water status, and Pi concentration. - Down-regulation of aldehyde oxidase expression and induced ABA signal transduction gene expression (*D-myo-inositol-3-phosphate synthase* and *14-3-3-like protein GF14*).	[74]
	Wheat (*Triticum Aestivum*)	*R. intraradices*	- Improved leaf area, RWC, and WUE. - Enhanced N and P content and grain yielding.	[75]
	Maize (*Zea mays*)	*Rhizophagus irregularis*	- Ameliorated PSII efficiency and membrane stability and decreased lipids’ oxidative damage. -TSS overaccumulation and aquaporin gene expression (down-regulated *ZmPIP1;1*, *ZmPIP1;3*, *ZmPIP1;4*, *ZmPIP1;6*, *ZmPIP2;2*, *ZmPIP2;4*, *ZmTIP1;1*, and *ZmTIP2;3* and up-regulated *ZmTIP4;1).*	[34]
	Sorghum (*Sorghum bicolor*)	*F. mosseae*	- Improved biomass and SLA and extended plant lifetime duration.	[47]
	Finger Millet (*Eleusinecoracana*)	*R. intraradices*	- Decreased EL, MDA, and hydrogen peroxide content. - Improved PRO, TSS, total phenol, and flavonoid content and antioxidant enzyme activities.	[50]
	Sorghum (*Sorghum bicolor*)	*R. arabicus*, *R. irregularis*	- Ameliorated transpiration efficiency and N and P acquisition.	[44]
	Wheat (*Triticum Aestivum*)	*Glomus intraradices*	- Improved RWC, flag leaf SLA, WUE, and mineral assimilation, particularly P.	[76]
	Maize (*Zea mays*)	*G. versiforme*	- Improved PH, SDM, and chlorophyll content. - Enhanced PRO, glycine betaine, TSS, free AAa, and phenols. - Decreased stress marker levels and improved GSH, CAT, POX, and SOD activities.	[51]
	Wheat (*Triticum aestivum*)	*F. mosseae*	- Enhanced SFW and SDW, N in roots, C/N ratio, and WUE. - Regulated plant secondary, oxidative stress metabolisms, and phytohormones’ crosstalk.	[35]
	Wheat (*Triticum aestivum*)	*R. irregularis*, *F. mosseae*	- Improved growth traits and N assimilation. - Regulated miR167, miR5384-3p, and miR156e-3p, influencing trafficking functionalities and cellular redox homeostasis.	[77]
	Maize (*Zea mays*)	*R. irregularis*	- Enhanced SFW and Pi acquisition - Moderated g_s_, transpiration, and WUE.	[45]
	Wheat (*Triticum aestivum*)	*R. intraradices*, *F. mosseae*, *F. geosporum*	- Improved plant and soil RWC. - Improved PSI and PSII quantum efficiency and photochemistry.	[43]
	Barley (*Hordeum vulagre*)	*R. intraradices*, *F. mossea*, *C. claroideum*,	- Improved plant growth traits.	[78]
	Rice (*Oryza sativa*)	*F. mosseae*, *F. geosporus*, *R. irregularis*, *G.microaggregatum*, *C. claroideum*	- Maintained rice growth and improved P acquisition. - Ameliorated nutrient, ABA, and IAA balance. - Higher grain yield, Chl fluorescence, and g_s_.	[42]
	Wheat (*Triticum aestivum*)	*F. mosseae*	- Enhanced plant shoot, root, and spike FW. - De-regulated transcriptional profiling, cell wall, and its membrane components. - Induced carbohydrate and lipid metabolism, cellulose synthase activity, membrane transport systems, N compound metabolic, and chitinase activity genes’ expression.	[37]
	Sorghum (*Sorghum bicolor*)	*G. mosseae*	- Ameliorated Chl a, b, and total Chl content, WUE, RWC, N, soluble proteins, and proline. - Enhanced yield, panicle length, number of panicles per plant, number of grains per panicle, and 1000-grain weight, and reduced EL and water saturation.	[36]
	Maize (*Zea mays*)	*F. mosseae*	- Enhanced Chl content, the net rate of photosynthesis, gs, rate of transpiration, and WUE.	[46]
	Wheat (*Triticum aestivum*)	*F. mosseae*	- Up-regulated water stress response-related genes (*TdsHN1* and *TdDRF1*).	[79]
Salinity				
	Maize (*Zea mays*)	*G. etunicatum*	- Improved dry biomass and nutrient content and decreased Na^+^ assimilation.	[60]
	Wheat (*Triticum aestivum*)	*G. intraradices*	- Reduced shoot Na^+^ and enhanced N, P, K^+^, proline, Chl, protein, SA content, and total grain yielding.	[58]
	Rice (*Oryza sativa*)	*C. etunicatum*	- Improved SDW and root length. - Ameliorated g_s_ and PSII efficiency, P and K^+^ content, K^+^/Na^+^ in shoots and reduced it in the roots. - Up-regulated *OsNHX3*, *OsSOS1*, *OsHKT2;1*, and *OsHKT1;5*.	[63]
	Maize (*Zea mays*)	*R. intraradices*	- Improved SDW and mineral uptake (P and N) and reduced leaf proline levels.	[52]
	Maize (*Zea mays*)	*C. lamellosum Gigaspora margarita*	- Ameliorated SDW, RDW, and nutrient content. - Reduced proline in the shoots and Na^+^ in the roots and higher K^+^/Na^+^ in roots. - Higher *ZmAKT2*, *ZmSOS1*, and *ZmSKOR* gene expression, sustaining K^+^ and Na^+^ homeostasis.	[61]
	Sorghum (*Sorghum bicolor*)	*Acaulospora mellea*	- Increased biomass, minerals, K^+^/Na^+^, leaf TSS content, and SOD, POX, CAT activities.	[67]
	Wheat (*Triticum aestivum*)	*R. irregularis*, *F. mosseae*, *F. geosporum*, *C. claroideum*	- Upgraded net photosynthesis rate, g_s_, reduced intrinsic WUE. - Higher carbon use efficiency and grain yielding.	[53]
	Maize (*Zea mays*)	*R. irregulare*	- Decreased Na^+^ level in root and its roots-to-shoots translocation. - K^+^ accumulation and Mg^2+^ reduction in roots. - Ca^2+^ fluctuation interacting with salinity.	[62]
	Rice (*Oryza sativa*)	*R. irregularis*	- Reduced H_2_O_2_ and enhanced CAT activity and leaf N content. - Enhanced tiller, panicle, grain number, and yield.	[54]
	Rice (*Oryza sativa*)	*F. mosseae*, *A. laevis*, *G. margarita*	- Improved total Chl, shoot K^+^/Na^+^ ratio, and grain yield. - Decreased shoot Na^+^/root Na^+^.	[64]
	Rice (*Oryza sativa*)	*G. etunicatum*, *G. geosporum*, *G. mosseae*	- Lower Na^+^/K^+^ ratio. - Maintained sucrose in flag leaf tissues and fructose and free proline overaccumulation. - Regulated cyanidin-3-glucoside and peonidin-3-glucosi.de in salt-sensitive rice.	[65]
	Maize (*Zea mays*)	*F. mosseae*	- Improved WUE, Chl, A_n_, CAT, SOD, APX, and GSH activities - Decreased EL, MDA, and H_2_O_2_.	[80]
	Wheat (*Triticum aestivum*)	*F. geosporum*, *F. mosseae*, *R. clarus*, *Scutellospora persica*	- Promoted N, P, and K^+^ acquisition, Chl content, and K^+^/Na^+^ ratio. - Reduced Na^+^, Cl^−^, PRO, and MDA content.	[59]
	Maize (*Zea mays*)	*G. mosseae*	- Improved PH, leaf number, and plant FW and DW. - Enhanced nutrient content (N, P, and K^+^), and increased antioxidant enzyme activities. - Improved palmitoleic, oleic, linoleic, and linolenic acid contents.	[56]
	Sorghum (*Sorghum bicolor*)	*F. mosseae*, *F. geosporum*	- Improved PH, FW, DW, P, K^+^/Na^+^, and glomalin in soil. - Enhanced dehydrogenase and alkaline phosphatase activity.	[66]
	Maize (*Zea mays*)	*R. irregularis*	*-* Improved SDW, RDW, and RWC.	[57]
Heavy Metals				
	Wheat (*Triticum aestivum*)	*Glomus* sp.	*-* Reduced Zn content in shoots.	[81]
	Rice (*Oryza sativa*)	*R. intraradices*, *F. mosseae*	*-* Reduced Cd concentration in shoots and roots.	[31]
	Maize (*Zea mays*)	*F. mosseae*, *Diversispora sphurcum*	- Enhanced biomass, Chl, SOD, and CAT activities, and T-AOC and reduced H_2_O_2_, and MDA levels. - Limited Pb, Zn, and Cd transfer and contents in shoots.	[71]
	Rice (*Oryza sativa*)	*F. mosseae*, *R. intraradices*	- Reduced Cd concentrations in shoots and roots. - Iduced *Nramp5* and *HMA3* expression in roots.	[70]
	Sorghum (*Sorghum bicolor*)	*C. etunicatum*	- Enhanced biomass, PSII efficiency, and P, N, and S assimilation, and Mo accumulation.	[72]
	Maize (*Zea mays*)	*C. etunicatum*	- Increased SDW and SFW, and K^+^, P, Ca^2+^, Mg^2+^ in shoots. - Reduced lanthanum concentration in shoot and root.	[69]
	Wheat (*Triticum aestivum*)	*R. intraradices*	*-* Ameliorated growth and reduced Ni assimilation.	[73]

AAs: amino acids; ABA: abscisic acid; An: net photosynthesis; C: carbon; Ca: calcium; CAT: catalase; Cd: cadmium; Chl: chlorophyll; Cl: chlorine; DW: dry weight; EL: electrolyte leakage; FW: fresh weight; POX: peroxidase; gs: stomatal conductance; GSH: glutathione; HKT: high-affinity K^+^ transporter; HMA: heavy metal ATPase; IAA: indole-3-acetic acid; K: potassium; MDA: malonyldialdehyde; Mg: magnesium; miR: microRNA; Mo: molybdene; N: nitrogen; Na: sodium; NHX: Na^+^/H^+^ exchangers; Ni: nikel; Nramp: natural resistance-associated macrophage protein; P: phosphorus; Pb: lead; PH: plant height; PIP: plasma membrane intrinsic protein; PRO: proline; PS: photosystem; RDW: root dry matter; RWC: relative water content; S: sulfur; SA: salicylic acid; SDW: shoot dry weight; SFW: shoot fresh weight; SLA: specific leaf area; SOD: superoxide dismutase; SOS: overly sensitive; T-AOC: total antioxidant capacity; TIP: tonoplast intrinsic protein; TSS: total soluble sugars; WUE: water use efficiency; Zn: zinc.

**Table 2:** AMF alleviate biotic stress in cereal crops.

AMF	Biotic Stress Agent	Plant	AMF Colonization Effects	Ref.
*F. mosseae* *R. irregularis*	*Blumeria graminis* f. sp. *tritici*	Wheat (*Triticum aestivum*)	- Reduced pathogenic fungus’ conidia number. - Overaccumulation of polyphenolic compounds.	[82]
*C. claroideum*, *F. mosseae*, *R. irregulare*	*Oscinella frit*	Wheat (*Triticum aestivum*)	- Ameliorated plant health status and production under pest’s heavy infestation.	[86]
*F. mosseae*	*B. graminis* f. sp. *tritici*	Wheat (*Triticum aestivum*)	- Decreased foliar biotrophic pathogen infection. - ISR and phenolic components’ overproduction.	[83]
*F. mosseae*	*Xanthomonas translucens*	Wheat (*Triticum aestivum*)	- Higher biomass, yield, and protein oxidation levels and decreased lesion area. - Up-regulated CYP enzymes, *NTRs* genes, and disease resilience genes.	[84]
*R. intraradices*	*Magnaporthe oryzae*	Rice (*Oryza sativa*)	- Induced IAA-/SA-related genes, key in pathogenesis-related protein synthesis. - Enriched JA, α-linolenic acid-, phenol-, and terpenoid syntheses-related genes.	[91]
*F. mosseae*, *R. intraradices*	*M. oryzae*	Rice (*Oryza sativa*)	- Improved Pi content and grain yield. - Higher resilience to the rice blast fungus.	[89]
*R. irregulare*	*Sitobion avenae*	Wheat (*Triticum aestivum*)	*-* Better harvest index and lower aphid population size.	[87]
*R. intraradices*	*Spodoptera frugiperda*	Rice (*Oryza sativa*)	- ISR activation and higher PPO and POX activity.	[92]
*Enthropospora* sp., *Gigaspora* sp., *Glomus* sp.	*Peronosclerospora* spp.	Maize (*Zea mays*)	- Enhanced SDM. - Attenuated downy mildew. - Expanded incubation period.	[90]
*R. intraradices*	*Fusarium pseudograminearum*	Wheat (*Triticum aestivum*)	- Improved biomass, spikes number, and height. - Enhanced antioxidant enzyme activity and reduced lipid peroxidation levels. - Decreased *F. pseudograminearum* density (76%) and disease severity (40%).	[88]
*F. mosseae*	*X. translucens*	Wheat (*Triticum aestivum*)	- Higher N acquisition, photosynthesis, and glucose and amino acid content. - Elicited defense-related proteins, immune response, and JA biosynthesis.	[85]
*R. irregularis*	*Rhopalosiphum padi*	Wheat (*Triticum aestivum*)	- Boosted root growth and P and N assimilation.	[93]

CYP: cytochrome P450; IAA: indole-3-acetic acid; ISR: induced systemic resistance; JA: jasmonic acid; N: nitrogen; NTR: nitrate/chlorate transporters; P: phosphorus; POX: peroxidase; PPO: polyphenol oxidase; SA: salicylic acid; SDM: shoot dry matter.

**Table 4:** AMF alleviate biotic stress in oilseed crops.

AMF	Biotic Stress Agent	Plant	AMF Colonization Effects	Ref.
*R. intraradices*, *G. mosseae*, *G. etunicatum*, *G. claroideum*, *G. microaggregatum*, *G. geosporum*	*Sclerotinia sclerotiorum*	Sunflower (*Helianthus annuus*)	- Hinder pathogenic hyphae development. - Localized and systemic resistance to white rot.	[120]
*R. intraradices*	*Macrophomina phaseolina*	Soybean (*Glycine max)*	- Reduced charcoal rot in soybean.	[119]
*R. irregularis*	*M. phaseolina*	Soybean *(Glycine max)*	- Up-regulated secondary metabolism genes. - Repressed genes encoding fasciclin-like arabinogalactan-protein, SKU5 similar 5, endo-chitinase, *MYB*, and POX.	[121]
*R. irregularis*	*F. virguliforme*	Soybean (*Glycine max*)	- Up-regulated defense-related genes like disease resistance proteins, *WRKY*, auxins, receptor kinases, proteases, thaumatin-like protein, pleiotropic drug resistance proteins/genes. - Down-regulated cell wall and *POX* genes.	[115]
*R. intraradices*	*F. virguliforme*	Soybean (*Glycine max*)	- Improved growth and P, K, Na, and S. - Decreased death syndrome severity.	[116]
*R. intraradices*	*M. phaseolina*	Soybean (*Glycine max*)	*-* Ameliorated biomass and greenness index.	[114]
*G. etunicatum*	*Heterodera glycines*	Soybean (*Glycine max)*	- Improved plant height and root system. - Decreased female nematodes in roots.	[117]
*R. irregularis*	*Aphis glycines*	Soybean (*Glycine max*)	*-* Improved biomass, nodulation, and N and C content.	[118]

C: carbon; K: potassium; N: nitrogen; Na: sodium; P: phosphorus; POX: peroxidase; S: sulfur; SKU: skewed-growth proteins.

## References

[1] Khoury C.K., Jarvis A., Jones A.D. (2020). Trade and Its Trade-Offs in the Food System. Nat. Food.

[2] Fones H.N., Bebber D.P., Chaloner T., Kay W.T., Steinberg G., Gurr S.J. (2020). Threats to Global Food Security from Emerging Fungal and Oomycete Crop Pathogens. Nat. Food.

[3] Cogato A., Meggio F., De Antoni Migliorati M., Marinello F. (2019). Extreme Weather Events in Agriculture: A Systematic Review. Sustainability.

[4] Wang J.-W., Luo Z.-H., Xu W., Ding J.-F., Zheng T.-L. (2016). Transformation of Dimethyl Phthalate Esters (DMPEs) by a Marine Red Yeast *Rhodotorula Mucilaginosa* Isolated from Deep Sea Sediments of the Atlantic Ocean. Int. Biodeterior. Biodegrad..

[5] Zhu J.-K. (2016). Abiotic Stress Signaling and Responses in Plants. Cell.

[6] Zhang W., Sun K., Shi R.-H., Yuan J., Wang X.-J., Dai C.-C. (2018). Auxin Signalling of *Arachis hypogaea* Activated by Colonization of Mutualistic Fungus *Phomopsis liquidambari* Enhances Nodulation and N_2_-Fixation: Auxin in Fungus-Legume-Rhizobium Interactions. Plant Cell Environ..

[7] Grassini P., Eskridge K.M., Cassman K.G. (2013). Distinguishing between Yield Advances and Yield Plateaus in Historical Crop Production Trends. Nat. Commun..

[8] Smith S.E., Read D. (2008). Mineral Nutrition, Toxic Element Accumulation and Water Relations of Arbuscular Mycorrhizal Plants. Mycorrhizal Symbiosis.

[9] Genre A., Lanfranco L., Perotto S., Bonfante P. (2020). Unique and Common Traits in Mycorrhizal Symbioses. Nat. Rev. Microbiol..

[10] Shi J., Wang X., Wang E. (2023). Mycorrhizal Symbiosis in Plant Growth and Stress Adaptation: From Genes to Ecosystems. Annu. Rev. Plant Biol..

[11] Huang D., Ma M., Wang Q., Zhang M., Jing G., Li C., Ma F. (2020). Arbuscular Mycorrhizal Fungi Enhanced Drought Resistance in Apple by Regulating Genes in the MAPK Pathway. Plant Physiol. Biochem..

[12] Ait-El-Mokhtar M., Baslam M., Ben-Laouane R., Anli M., Boutasknit A., Mitsui T., Wahbi S., Meddich A. (2020). Alleviation of Detrimental Effects of Salt Stress on Date Palm (*Phoenix dactylifera* L.) by the Application of Arbuscular Mycorrhizal Fungi and/or Compost. Front. Sustain. Food Syst..

[13] Anli M., Baslam M., Tahiri A., Raklami A., Symanczik S., Boutasknit A., Ait-El-Mokhtar M., Ben-Laouane R., Toubali S., Ait Rahou Y. (2020). Biofertilizers as Strategies to Improve Photosynthetic Apparatus, Growth, and Drought Stress Tolerance in the Date Palm. Front. Plant Sci..

[14] Begum N., Qin C., Ahanger M.A., Raza S., Khan M.I., Ashraf M., Ahmed N., Zhang L. (2019). Role of Arbuscular Mycorrhizal Fungi in Plant Growth Regulation: Implications in Abiotic Stress Tolerance. Front. Plant Sci..

[15] Wahab A., Muhammad M., Munir A., Abdi G., Zaman W., Ayaz A., Khizar C., Reddy S.P.P. (2023). Role of Arbuscular Mycorrhizal Fungi in Regulating Growth, Enhancing Productivity, and Potentially Influencing Ecosystems under Abiotic and Biotic Stresses. Plants.

[16] Ben-Laouane R., Baslam M., Ait-El-Mokhtar M., Anli M., Boutasknit A., Ait-Rahou Y., Toubali S., Mitsui T., Oufdou K., Wahbi S. (2020). Potential of Native Arbuscular Mycorrhizal Fungi, Rhizobia, and/or Green Compost as Alfalfa (*Medicago sativa*) Enhancers under Salinity. Microorganisms.

[17] Frew A. (2021). Contrasting Effects of Commercial and Native Arbuscular Mycorrhizal Fungal Inoculants on Plant Biomass Allocation, Nutrients, and Phenolics. Plants People Planet.

[18] Smith G.R., Finlay R.D., Stenlid J., Vasaitis R., Menkis A. (2017). Growing Evidence for Facultative Biotrophy in Saprotrophic Fungi: Data from Microcosm Tests with 201 Species of Wood-decay Basidiomycetes. New Phytol..

[19] Dowarah B., Gill S.S., Agarwala N. (2022). Arbuscular Mycorrhizal Fungi in Conferring Tolerance to Biotic Stresses in Plants. J. Plant Growth Regul..

[20] Bahadur A., Batool A., Nasir F., Jiang S., Mingsen Q., Zhang Q., Pan J., Liu Y., Feng H. (2019). Mechanistic Insights into Arbuscular Mycorrhizal Fungi-Mediated Drought Stress Tolerance in Plants. Int. J. Mol. Sci..

[21] Bhatta M., Morgounov A., Belamkar V., Wegulo S.N., Dababat A.A., Erginbas-Orakci G., El Bouhssini M., Gautam P., Poland J., Akci N. (2019). Genome-Wide Association Study for Multiple Biotic Stress Resistance in Synthetic Hexaploid Wheat. Int. J. Mol. Sci..

[22] Zheng F.L., Liang S.M., Chu X.N., Yang Y.L., Wu Q.S. (2020). Mycorrhizal Fungi Enhance Flooding Tolerance of Peach through Inducing Proline Accumulation and Improving Root Architecture. Plant Soil Environ..

[23] Meddich A. (2022). Biostimulants for Resilient Agriculture—Improving Plant Tolerance to Abiotic Stress: A Concise Review. Gesunde Pflanz..

[24] Diagne N., Ngom M., Djighaly P.I., Fall D., Hocher V., Svistoonoff S. (2020). Roles of Arbuscular Mycorrhizal Fungi on Plant Growth and Performance: Importance in Biotic and Abiotic Stressed Regulation. Diversity.

[25] Halford N.G., Curtis T.Y., Chen Z., Huang J. (2015). Effects of Abiotic Stress and Crop Management on Cereal Grain Composition: Implications for Food Quality and Safety. J. Exp. Bot..

[26] Chikkaputtaiah C., Debbarma J., Baruah I., Havlickova L., Deka Boruah H.P., Curn V. (2017). Molecular Genetics and Functional Genomics of Abiotic Stress-Responsive Genes in Oilseed Rape (*Brassica napus* L.): A Review of Recent Advances and Future. Plant Biotechnol. Rep..

[27] Slimani A., Ait-El-Mokhtar M., Ben-Laouane R., Boutasknit A., Anli M., Abouraicha E.F., Oufdou K., Meddich A., Baslam M. (2024). Molecular and Systems Biology Approaches for Harnessing the Symbiotic Interaction in Mycorrhizal Symbiosis for Grain and Oil Crop Cultivation. Int. J. Mol. Sci..

[28] Lenoir I., Fontaine J., Lounès-Hadj Sahraoui A. (2016). Arbuscular Mycorrhizal Fungal Responses to Abiotic Stresses: A Review. Phytochemistry.

[29] Sharma N., Yadav K., Cheema J., Badda N., Aggarwal A. (2015). Arbuscular mycorrhizal symbiosis and water stress: A critical review. Pertanika J. Trop. Agric. Sci..

[30] Zhao R., Guo W., Bi N., Guo J., Wang L., Zhao J., Zhang J. (2015). Arbuscular Mycorrhizal Fungi Affect the Growth, Nutrient Uptake and Water Status of Maize (*Zea mays* L.) Grown in Two Types of Coal Mine Spoils under Drought Stress. Appl. Soil Ecol..

[31] Li H., Luo N., Zhang L.J., Zhao H.M., Li Y.W., Cai Q.Y., Wong M.H., Mo C.H. (2016). Do Arbuscular Mycorrhizal Fungi Affect Cadmium Uptake Kinetics, Subcellular Distribution and Chemical Forms in Rice?. Sci. Total. Environ..

[32] Zhang Y.C., Wang P., Wu Q.H., Zou Y.N., Bao Q., Wu Q.S. (2017). Arbuscular Mycorrhizas Improve Plant Growth and Soil Structure in Trifoliate Orange under Salt Stress. Arch. Agron. Soil Sci..

[33] Zhang F., Zou Y.N., Wu Q.S. (2018). Quantitative Estimation of Water Uptake by Mycorrhizal Extraradical Hyphae in Citrus under Drought Stress. Sci. Hortic..

[34] Quiroga G., Erice G., Aroca R., Chaumont F., Ruiz-Lozano J.M. (2017). Enhanced Drought Stress Tolerance by the Arbuscular Mycorrhizal Symbiosis in a Drought-Sensitive Maize Cultivar Is Related to a Broader and Differential Regulation of Host Plant Aquaporins than in a Drought-Tolerant Cultivar. Front. Plant Sci..

[35] Bernardo L., Carletti P., Badeck F.W., Rizza F., Morcia C., Ghizzoni R., Rouphael Y., Colla G., Terzi V., Lucini L. (2019). Metabolomic Responses Triggered by Arbuscular Mycorrhiza Enhance Tolerance to Water Stress in Wheat Cultivars. Plant Physiol. Biochem..

[36] Kamali S., Mehraban A. (2021). Effects of Nitroxin and Arbuscular Mycorrhizal Fungi on the Agro-Physiological Traits and Grain Yield of Sorghum (*Sorghum bicolor* L.) under Drought Stress Conditions. PLoS ONE.

[37] Moradi Tarnabi Z., Iranbakhsh A., Mehregan I., Ahmadvand R. (2020). Impact of Arbuscular Mycorrhizal Fungi (AMF) on Gene Expression of Some Cell Wall and Membrane Elements of Wheat (*Triticum aestivum* L.) under Water Deficit Using Transcriptome Analysis. Physiol. Mol. Biol. Plants.

[38] Gutjahr C. (2014). Phytohormone Signaling in Arbuscular Mycorhiza Development. Curr. Opin. Plant Biol..

[39] Etemadi M., Gutjahr C., Couzigou J.M., Zouine M., Lauressergues D., Timmers A., Audran C., Bouzayen M., Bécard G., Combier J.P. (2014). Auxin Perception Is Required for Arbuscule Development in Arbuscular Mycorrhizal Symbiosis. Plant Physiol..

[40] Martín-Rodríguez J.Á., León-Morcillo R., Vierheilig H., Ocampo J.A., Ludwig-Müller J., García-Garrido J.M. (2011). Ethylene-Dependent/Ethylene-Independent ABA Regulation of Tomato Plants Colonized by Arbuscular Mycorrhiza Fungi. New Phytol..

[41] Foo E., Ross J.J., Jones W.T., Reid J.B. (2013). Plant Hormones in Arbuscular Mycorrhizal Symbioses: An Emerging Role for Gibberellins. Ann. Bot..

[42] Chareesri A., De Deyn G.B., Sergeeva L., Polthanee A., Kuyper T.W. (2020). Increased Arbuscular Mycorrhizal Fungal Colonization Reduces Yield Loss of Rice (*Oryza sativa* L.) under Drought. Mycorrhiza.

[43] Mathur S., Tomar R.S., Jajoo A. (2019). Arbuscular Mycorrhizal Fungi (AMF) Protects Photosynthetic Apparatus of Wheat under Drought Stress. Photosynth. Res..

[44] Symanczik S., Lehmann M.F., Wiemken A., Boller T., Courty P.-E. (2018). Effects of Two Contrasted Arbuscular Mycorrhizal Fungal Isolates on Nutrient Uptake by *Sorghum bicolor* under Drought. Mycorrhiza.

[45] Le Pioufle O., Ganoudi M., Calonne-salmon M., Dhaou F.B., Declerck S. (2019). *Rhizophagus irregularis* MUCL 41833 Improves Phosphorus Uptake and Water Use Efficiency in Maize Plants During Recovery From Drought Stress. Front. Plant Sci..

[46] Sun J., Yang L., Yang X., Wei J., Li L., Guo E., Kong Y. (2021). Using Spectral Reflectance to Estimate the Leaf Chlorophyll Content of Maize Inoculated With Arbuscular Mycorrhizal Fungi Under Water Stress. Front. Plant Sci..

[47] Sun X., Shi J., Ding G. (2017). Combined Effects of Arbuscular Mycorrhiza and Drought Stress on Plant Growth and Mortality of Forage Sorghum. Appl. Soil Ecol..

[48] Pan J., Huang C., Peng F., Zhang W., Luo J., Ma S., Xue X. (2020). Effect of Arbuscular Mycorrhizal Aungi (AMF) and Plant Growth-Promoting Bacteria (PGPR) Inoculations on *Elaeagnus angustifolia* L. in Saline Soil. Appl. Sci..

[49] Bitterlich M., Franken P., Graefe J. (2019). Atmospheric Drought and Low Light Impede Mycorrhizal Effects on Leaf Photosynthesis —A Glasshouse Study on Tomato under Naturally Fluctuating Environmental Conditions. Mycorrhiza.

[50] Tyagi J., Varma A., Pudake R.N. (2017). Evaluation of Comparative Effects of Arbuscular Mycorrhiza (*Rhizophagus intraradices*) and Endophyte (*Piriformospora indica*) Association with Finger Millet (*Eleusine coracana*) under Drought Stress. Eur. J. Soil Biol..

[51] Begum N., Ahanger M.A., Su Y., Lei Y., Mustafa N.S.A., Ahmad P., Zhang L. (2019). Improved Drought Tolerance by AMF Inoculation in Maize (*Zea mays*) Involves Physiological and Biochemical Implications. Plants.

[52] Krishnamoorthy R., Kim K., Subramanian P., Senthilkumar M., Anandham R., Sa T. (2016). Arbuscular Mycorrhizal Fungi and Associated Bacteria Isolated from Salt-Affected Soil Enhances the Tolerance of Maize to Salinity in Coastal Reclamation Soil. Agric. Ecosyst. Environ..

[53] Eroğlu Ç.G., Cabral C., Ravnskov S., Bak Topbjerg H., Wollenweber B. (2020). Arbuscular Mycorrhiza Influences Carbon-use Efficiency and Grain Yield of Wheat Grown under Pre- and Post-anthesis Salinity Stress. Plant Biol..

[54] Norouzinia F., Ansari M.H., Aminpanah H., Firozi S. (2020). Alleviation of Soil Salinity on Physiological and Agronomic Traits of Rice Cultivars Using Arbuscular Mycorrhizal Fungi and Pseudomonas Strains Under Field Conditions. Rev. Agric. Neotrop..

[55] Wang G., Kong Y., Liu Y., Li D., Zhang X., Yuan J., Li G. (2020). Evolution of Phytotoxicity during the Active Phase of Co-Composting of Chicken Manure, Tobacco Powder and Mushroom Substrate. Waste Manag..

[56] Ndiate N.I., Saeed Q., Haider F.U., Liqun C., Nkoh J.N., Mustafa A. (2021). Co-Application of Biochar and Arbuscular Mycorrhizal Fungi Improves Salinity Tolerance, Growth and Lipid Metabolism of Maize (*Zea mays* L.) in an Alkaline Soil. Plants.

[57] Chen Q., Deng X., Elzenga J.T.M., van Elsas J.D. (2022). Effect of Soil Bacteriomes on Mycorrhizal Colonization by *Rhizophagus irregularis*—Interactive Effects on Maize (*Zea mays* L.) Growth under Salt Stress. Biol. Fertil. Soils.

[58] Aboul-Nasr A., Al-Fayoumy M., Hussein M., Elhabbab A. (2016). Potential Application of *Glomus Intraradices* (AMF) and Different Isolates of PGPR (Biotol) to Enhance the Yield and Quality of Wheat Grown in The Field in Calcareous Soil Under Different Salinity Levels. J. Adv. Agric. Res..

[59] Elgharably A., Nafady N.A. (2021). Inoculation with Arbuscular Mycorrhizae, *Penicillium funiculosum* and *Fusarium oxysporum* Enhanced Wheat Growth and Nutrient Uptake in the Saline Soil. Rhizosphere.

[60] Lee Y., Krishnamoorthy R., Selvakumar G., Kim K., Sa T. (2015). Alleviation of Salt Stress in Maize Plant by Co-Inoculation of Arbuscular Mycorrhizal Fungi and *Methylobacterium oryzae* CBMB20. J. Korean Soc. Appl. Biol. Chem..

[61] Selvakumar G., Shagol C.C., Kim K., Han S., Sa T. (2018). Spore Associated Bacteria Regulates Maize Root K^+^/Na^+^ Ion Homeostasis to Promote Salinity Tolerance during Arbuscular Mycorrhizal Symbiosis. BMC Plant Biol..

[62] Moreira H., Pereira S.I.A., Vega A., Castro P.M.L., Marques A.P.G.C. (2020). Synergistic Effects of Arbuscular Mycorrhizal Fungi and Plant Growth-Promoting Bacteria Benefit Maize Growth under Increasing Soil Salinity. J. Environ. Manag..

[63] Porcel R., Aroca R., Azcon R., Ruiz-Lozano J.M. (2016). Regulation of Cation Transporter Genes by the Arbuscular Mycorrhizal Symbiosis in Rice Plants Subjected to Salinity Suggests Improved Salt Tolerance Due to Reduced Na^+^ Root-to-Shoot Distribution. Mycorrhiza.

[64] Parvin S., Van Geel M., Yeasmin T., Verbruggen E., Honnay O. (2020). Effects of Single and Multiple Species Inocula of Arbuscular Mycorrhizal Fungi on the Salinity Tolerance of a Bangladeshi Rice (*Oryza sativa* L.) Cultivar. Mycorrhiza.

[65] Tisarum R., Theerawitaya C., Samphumphuang T., Polispitak K., Thongpoem P., Singh H.P., Cha-um S. (2020). Alleviation of Salt Stress in Upland Rice (*Oryza sativa* L. Ssp. *Indica* Cv. Leum Pua) Using Arbuscular Mycorrhizal Fungi Inoculation. Front. Plant Sci..

[66] Chandra P., Singh A., Prajapat K., Rai A.K., Yadav R.K. (2022). Native Arbuscular Mycorrhizal Fungi Improve Growth, Biomass Yield, and Phosphorus Nutrition of Sorghum in Saline and Sodic Soils of the Semi–Arid Region. Environ. Exp. Bot..

[67] Wang F., Sun Y., Shi Z. (2019). Arbuscular Mycorrhiza Enhances Biomass Production and Salt Tolerance of Sweet Sorghum. Microorganisms.

[68] Lehmann J., Hansel C.M., Kaiser C., Kleber M., Maher K., Manzoni S., Nunan N., Reichstein M., Schimel J.P., Torn M.S. (2020). Persistence of Soil Organic Carbon Caused by Functional Complexity. Nat. Geosci..

[69] Hao L., Zhang Z., Hao B., Diao F., Zhang J., Bao Z., Guo W. (2021). Arbuscular Mycorrhizal Fungi Alter Microbiome Structure of Rhizosphere Soil to Enhance Maize Tolerance to La. Ecotoxicol. Environ. Saf..

[70] Chen X.W., Wu L., Luo N., Mo C.H., Wong M.H., Li H. (2019). Arbuscular Mycorrhizal Fungi and the Associated Bacterial Community Influence the Uptake of Cadmium in Rice. Geoderma.

[71] Zhan F., Li B., Jiang M., Yue X., He Y., Xia Y., Wang Y. (2018). Arbuscular Mycorrhizal Fungi Enhance Antioxidant Defense in the Leaves and the Retention of Heavy Metals in the Roots of Maize. Environ. Sci. Pollut. Res..

[72] Shi Z., Zhang J., Lu S., Li Y., Wang F. (2020). Arbuscular Mycorrhizal Fungi Improve the Performance of Sweet Sorghum Grown in a Mo-Contaminated Soil. J. Fungi.

[73] Rehman S., Mansoora N., Al-Dhumri S.A., Amjad S.F., Al-Shammari W.B., Almutari M.M., Alhusayni F.S., Al Bakre D.A., Lalarukh I., Alshahri A.H. (2022). Associative Effects of Activated Carbon Biochar and Arbuscular Mycorrhizal Fungi on Wheat for Reducing Nickel Food Chain Bioavailability. Environ. Technol. Innov..

[74] Li T., Sun Y., Ruan Y., Xu L., Hu Y., Hao Z., Zhang X., Li H., Wang Y., Yang L. (2016). Potential Role of *D-Myo-Inositol-3-Phosphate Synthase* and *14-3-3* Genes in the Crosstalk between *Zea mays* and *Rhizophagus intraradices* under Drought Stress. Mycorrhiza.

[75] Zhang Y., Malzahn A.A., Sretenovic S., Qi Y. (2019). The Emerging and Uncultivated Potential of CRISPR Technology in Plant Science. Nat. Plants.

[76] Zhang B., Zhang H., Wang H., Wang P., Wu Y., Wang M. (2018). Effect of Phosphorus Additions and Arbuscular Mycorrhizal Fungal Inoculation on the Growth, Physiology, and Phosphorus Uptake of Wheat Under Two Water Regimes. Commun. Soil Sci. Plant Anal..

[77] Fileccia V., Ingraffia R., Amato G., Giambalvo D., Martinelli F. (2019). Identification of microRNAS Differentially Regulated by Water Deficit in Relation to Mycorrhizal Treatment in Wheat. Mol. Biol. Rep..

[78] Sendek A., Karakoç C., Wagg C., Domínguez-Begines J., do Couto G.M., van der Heijden M.G.A., Naz A.A., Lochner A., Chatzinotas A., Klotz S. (2019). Drought Modulates Interactions between Arbuscular Mycorrhizal Fungal Diversity and Barley Genotype Diversity. Sci. Rep..

[79] Fiorilli V., Maghrebi M., Novero M., Votta C., Mazzarella T., Buffoni B., Astolfi S., Vigani G. (2022). Arbuscular Mycorrhizal Symbiosis Differentially Affects the Nutritional Status of Two Durum Wheat Genotypes under Drought Conditions. Plants.

[80] Wang H., Liang L., Liu B., Huang D., Liu S., Liu R., Siddique K.H.M., Chen Y. (2020). Arbuscular Mycorrhizas Regulate Photosynthetic Capacity and Antioxidant Defense Systems to Mediate Salt Tolerance in Maize. Plants.

[81] Sadia K., Asma B., Riffat N.M. (2016). Role of Arbuscular Mycorrhizal Fungi in Phytoremediation of Heavy Metals and Effects on Growth and Biochemical Activities of Wheat (*Triticum aestivum* L.) Plants in Zn Contaminated Soils. Afr. J. Biotechnol..

[82] Mustafa G., Randoux B., Tisserant B., Fontaine J., Magnin-Robert M., Lounès-Hadj Sahraoui A., Reignault P. (2016). Phosphorus Supply, Arbuscular Mycorrhizal Fungal Species, and Plant Genotype Impact on the Protective Efficacy of Mycorrhizal Inoculation against Wheat Powdery Mildew. Mycorrhiza.

[83] Mustafa G., Khong N.G., Tisserant B., Randoux B., Fontaine J., Magnin-Robert M., Reignault P., Sahraoui A.L.H. (2017). Defence Mechanisms Associated with Mycorrhiza-Induced Resistance in Wheat against Powdery Mildew. Funct. Plant Biol..

[84] Fiorilli V., Vannini C., Ortolani F., Garcia-Seco D., Chiapello M., Novero M., Domingo G., Terzi V., Morcia C., Bagnaresi P. (2018). Omics Approaches Revealed How Arbuscular Mycorrhizal Symbiosis Enhances Yield and Resistance to Leaf Pathogen in Wheat. Sci. Rep..

[85] Vannini C., Domingo G., Fiorilli V., Seco D.G., Novero M., Marsoni M., Wisniewski-Dye F., Bracale M., Moulin L., Bonfante P. (2021). Proteomic Analysis Reveals How Pairing of a Mycorrhizal Fungus with Plant Growth-Promoting Bacteria Modulates Growth and Defense in Wheat. Plant Cell Environ..

[86] Imperiali N., Chiriboga X., Schlaeppi K., Fesselet M., Villacrés D., Jaffuel G., Bender S.F., Dennert F., Blanco-Pérez R., van der Heijden M.G.A. (2017). Combined Field Inoculations of *Pseudomonas* Bacteria, Arbuscular Mycorrhizal Fungi, and Entomopathogenic Nematodes and Their Effects on Wheat Performance. Front. Plant Sci..

[87] Pons C., Voß A.C., Schweiger R., Müller C. (2020). Effects of Drought and Mycorrhiza on Wheat and Aphid Infestation. Ecol. Evol..

[88] Spagnoletti F.N., Carmona M., Balestrasse K., Chiocchio V., Giacometti R., Lavado R.S. (2021). The Arbuscular Mycorrhizal Fungus *Rhizophagus intraradices* Reduces the Root Rot Caused by *Fusarium pseudograminearum* in Wheat. Rhizosphere.

[89] Campo S., Martín-Cardoso H., Olivé M., Pla E., Catala-Forner M., Martínez-Eixarch M., San Segundo B. (2020). Effect of Root Colonization by Arbuscular Mycorrhizal Fungi on Growth, Productivity and Blast Resistance in Rice. Rice.

[90] Prasetyo J., Ginting C., Akin H.M., Suharjo R., Niswati A., Afandi A., Adiwijaya R., Sudiono S., Nurdin M. (2021). The Effect of Biological Agent and Botanical Fungicides on Maize Downy Mildew. Biodiversitas.

[91] Tian L., Chang C., Ma L., Nasir F., Zhang J., Li W., Tran L.-S.P., Tian C. (2019). Comparative Study of the Mycorrhizal Root Transcriptomes of Wild and Cultivated Rice in Response to the Pathogen *Magnaporthe oryzae*. Rice.

[92] Bhavanam S., Stout M.J. (2021). Assessment of Silicon-and Mycorrhizae-Mediated Constitutive and Induced Systemic Resistance in Rice, *Oryza sativa* L., against the Fall Armyworm, *Spodoptera frugiperda* Smith. Plants.

[93] Charters M.D., Durant E.K., Sait S.M., Field K.J. (2022). Impacts of Aphid Herbivory on Mycorrhizal Growth Responses across Three Cultivars of Wheat. Plants People Planet.

[94] Liu Z., Li Y., Ma L., Wei H., Zhang J., He X., Tian C. (2015). Coordinated Regulation of Arbuscular Mycorrhizal Fungi and Soybean MAPK Pathway Genes Improved Mycorrhizal Soybean Drought Tolerance. Mol. Plant-Microbe Interact..

[95] Igiehon O.N., Babalola O.O. (2021). *Rhizobium* and Mycorrhizal Fungal Species Improved Soybean Yield Under Drought Stress Conditions. Curr. Microbiol..

[96] Sheteiwy M.S., Abd Elgawad H., Xiong Y.C., Macovei A., Brestic M., Skalicky M., Shaghaleh H., Alhaj Hamoud Y., El-Sawah A.M. (2021). Inoculation with *Bacillus amyloliquefaciens* and Mycorrhiza Confers Tolerance to Drought Stress and Improve Seed Yield and Quality of Soybean Plant. Physiol. Plant..

[97] Al-Karaki G.N., Williams M. (2021). Mycorrhizal Mixtures Affect the Growth, Nutrition, and Physiological Responses of Soybean to Water Deficit. Acta Physiol. Plant..

[98] Askari A., Ardakani M.R., Vazan S., Paknejad F., Hosseini Y. (2018). The Effect of Mycorrhizal Symbiosis and Seed Priming on the Amount of Chlorophyll Index and Absorption of Nutrients under Drought Stress in Sesame Plant under Field Conditions. Appl. Ecol. Environ. Res..

[99] Gholinezhad E., Darvishzadeh R. (2021). Field Crops Research Influence of Arbuscular Mycorrhiza Fungi and Drought Stress on Fatty Acids Profile of Sesame (*Sesamum indicum* L.). Field Crops Res..

[100] Rahimzadeh S., Pirzad A. (2017). Microorganisms (AMF and PSB) Interaction on Linseed Productivity under Water-Deficit Condition. Int. J. Plant Prod..

[101] Ansari A., Razmjoo J., Zarei M., Karimmojeni H. (2021). Salicylic Acid Affects Mycorrhizal Features, Antioxidant Enzyme Activities and Seed Yield of Linseed under Water—Deficit Stress in Open—Field Conditions. Biol. Futur..

[102] Oliveira T.C., Cabral J.S.R., Santana L.R., Tavares G.G., Santos L.D.S., Paim T.P., Müller C., Silva F.G., Costa A.C., Souchie E.L. (2022). The Arbuscular Mycorrhizal Fungus *Rhizophagus clarus* Improves Physiological Tolerance to Drought Stress in Soybean Plants. Sci. Rep..

[103] Ghasemi N., Gholamhoseini M., Bazrafshan F., Habibzadeh F. (2022). Yield, Irrigation Water Productivity and Nutrient Uptake of Arbuscular Mycorrhiza Inoculated Sesame under Drought Stress Conditions. Agric. Water Manag..

[104] Pereira S.I.A., Moreira H., Argyras K., Castro P.M.L., Marques A.P.G.C. (2016). Promotion of Sunflower Growth under Saline Water Irrigation by the Inoculation of Beneficial Microorganisms. Appl. Soil Ecol..

[105] Ghouchani R., Abbaspour H., Saed-Moucheshi A., Pessarakli M. (2017). Colonization with Endo-Mycorrhiza Affects the Resistance of Safflower in Response to Salinity Condition. J. Plant Nutr..

[106] Heydari S., Pirzad A. (2021). Efficiency of *Funneliformis mosseae* and *Thiobacillus* Sp. on the Secondary Metabolites (Essential Oil, Seed Oil and Mucilage) of *Lallemantia iberica* under Salinity Stress. J. Hortic. Sci. Biotechnol..

[107] Qin W., Yan H., Zou B., Guo R., Ci D., Tang Z., Zou X., Zhang X., Yu X., Wang Y. (2021). Arbuscular Mycorrhizal Fungi Alleviate Salinity Stress in Peanut: Evidence from Pot-Grown and Field Experiments. Food Energy Secur..

[108] Hashem A., Abd_Allah E.F., Alqarawi A.A., Wirth S., Egamberdieva D. (2019). Comparing Symbiotic Performance and Physiological Responses of Two Soybean Cultivars to Arbuscular Mycorrhizal Fungi under Salt Stress. Saudi J. Biol. Sci..

[109] Păun A., Neagoe A., Păun M., Baciu I., Iordache V. (2015). Heavy Metal-Induced Differential Responses to Oxidative Stress and Protection by Mycorrhization in Sunflowers Grown in Lab and Field Scales. Pol. J. Environ. Stud..

[110] Molina A.S., Lugo M.A., Pérez Chaca M.V., Vargas-Gil S., Zirulnik F., Leporati J., Ferrol N., Azcón-Aguilar C. (2020). Effect of Arbuscular Mycorrhizal Colonization on Cadmium-Mediated Oxidative Stress in *Glycine max* (L.) Merr. Plants.

[111] Adeyemi N.O., Atayese M.O., Sakariyawo O.S., Azeez J.O., Abayomi Sobowale S.P., Olubode A., Mudathir R., Adebayo R., Adeoye S. (2021). Alleviation of Heavy Metal Stress by Arbuscular Mycorrhizal Symbiosis in *Glycine max* L. Grown in Copper, Lead and Zinc Contaminated Soils. Rhizosphere.

[112] Sheteiwy M.S., Ali D.F.I., Xiong Y.C., Brestic M., Skalicky M., Hamoud Y.A., Ulhassan Z., Shaghaleh H., AbdElgawad H., Farooq M. (2021). Physiological and Biochemical Responses of Soybean Plants Inoculated with Arbuscular Mycorrhizal Fungi and Bradyrhizobium under Drought Stress. BMC Plant Biol..

[113] Hoang D.T.T., Rashtbari M., Anh L.T., Wang S., Tu D.T., Hiep N.V., Razavi B.S. (2022). Mutualistic Interaction between Arbuscular Mycorrhiza Fungi and Soybean Roots Enhances Drought Resistant through Regulating Glucose Exudation and Rhizosphere Expansion. Soil Biol. Biochem..

[114] Spagnoletti F.N., Cornero M., Chiocchio V., Lavado R.S., Roberts I.N. (2020). Arbuscular Mycorrhiza Protects Soybean Plants against *Macrophomina phaseolina* Even under Nitrogen Fertilization. Eur. J. Plant Pathol..

[115] Marquez N., Giachero M.L., Gallou A., Debat H.J., Declerck S., Ducasse D.A. (2019). Transcriptome Analysis of Mycorrhizal and Nonmycorrhizal Soybean Plantlets upon Infection with *Fusarium virguliforme*, One Causal Agent of Sudden Death Syndrome. Plant Pathol..

[116] Pawlowski M.L., Hartman G.L. (2020). Reduction of Sudden Death Syndrome Foliar Symptoms and *Fusarium virguliforme* DNA in Roots Inoculated with *Rhizophagus intraradices*. Plant Dis..

[117] Benedetti T., Antoniolli Z.I., Sordi E., Carvalho I.R., Bortoluzzi E.C. (2021). Use of the *Glomus etunicatum* as Biocontrol Agent of the Soybean Cyst Nematode. Res., Soc. Dev..

[118] Dabré É.E., Hijri M., Favret C. (2022). Influence on Soybean Aphid by the Tripartite Interaction between Soybean, a Rhizobium Bacterium, and an Arbuscular Mycorrhizal Fungus. Microorganisms.

[119] Spagnoletti F., Carmona M., Gómez N.E.T., Chiocchio V., Lavado R.S. (2017). Arbuscular Mycorrhiza Reduces the Negative Effects of *M. Phaseolina* on Soybean Plants in Arsenic-Contaminated Soils. Appl. Soil Ecol..

[120] Bán R., Baglyas G., Virányi F., Barna B., Posta K., Kiss J., Körösi K. (2017). The Chemical Inducer, BTH (Benzothiadiazole) and Root Colonization by Mycorrhizal Fungi (*Glomus* Spp.) Trigger Resistance against White Rot (*Sclerotinia sclerotiorum*) in Sunflower. Acta Biol. Hung..

[121] Marquez N., Giachero M.L., Gallou A., Debat H.J., Cranenbrouck S., Di Rienzo J.A., Pozo M.J., Ducasse D.A., Declerck S. (2018). Transcriptional Changes in Mycorrhizal and Nonmycorrhizal Soybean Plants upon Infection with the Fungal Pathogen *Macrophomina phaseolina*. Mol. Plant-Microbe Interact..

[122] Quiroga G., Erice G., Ding L., Chaumont F., Aroca R., Ruiz-Lozano J.M. (2019). The Arbuscular Mycorrhizal Symbiosis Regulates Aquaporins Activity and Improves Root Cell Water Permeability in Maize Plants Subjected to Water Stress. Plant Cell Environ..

[123] Li T., Hu Y.J., Hao Z.P., Li H., Wang Y.S., Chen B.D. (2013). First Cloning and Characterization of Two Functional Aquaporin Genes from an Arbuscular Mycorrhizal Fungus *Glomus intraradices*. New Phytol..

[124] Quiroga G., Erice G., Aroca R., Chaumont F., Ruiz-Lozano J.M. (2019). Contribution of the Arbuscular Mycorrhizal Symbiosis to the Regulation of Radial Root Water Transport in Maize Plants under Water Deficit. Environ. Exp. Bot..

[125] Quiroga G., Erice G., Aroca R., Delgado-Huertas A., Ruiz-Lozano J.M. (2020). Elucidating the Possible Involvement of Maize Aquaporins and Arbuscular Mycorrhizal Symbiosis in the Plant Ammonium and Urea Transport under Drought Stress Conditions. Plants.

[126] Gong M., Bai N., Wang P., Su J., Chang Q., Zhang Q. (2023). Co-Inoculation with Arbuscular Mycorrhizal Fungi and Dark Septate Endophytes under Drought Stress: Synergistic or Competitive Effects on Maize Growth, Photosynthesis, Root Hydraulic Properties and Aquaporins?. Plants.

[127] Asadollahi M., Iranbakhsh A., Ahmadvand R., Ebadi M., Mehregan I. (2023). Synergetic Effect of Water Deficit and Arbuscular Mycorrhizal Symbiosis on the Expression of Aquaporins in Wheat (*Triticum aestivum* L.) Roots: Insights from NGS RNA-Sequencing. Physiol. Mol. Biol. Plants.

[128] Wipf D., Krajinski F., van Tuinen D., Recorbet G., Courty P.E. (2019). Trading on the Arbuscular Mycorrhiza Market: From Arbuscules to Common Mycorrhizal Networks. New Phytol..

[129] Calabrese S., Cusant L., Sarazin A., Niehl A., Erban A., Brulé D., Recorbet G., Wipf D., Roux C., Kopka J. (2019). Imbalanced Regulation of Fungal Nutrient Transports According to Phosphate Availability in a Symbiocosm Formed by Poplar, Sorghum, and *Rhizophagus irregularis*. Front. Plant Sci..

[130] Chen Z., Zhou M., Newman I.A., Mendham N.J., Zhang G., Shabala S. (2007). Potassium and Sodium Relations in Salinised Barley Tissues as a Basis of Differential Salt Tolerance. Funct. Plant Biol..

[131] Estrada B., Aroca R., Maathuis F.J.M., Barea J.M., Ruiz-Lozano J.M. (2013). Arbuscular Mycorrhizal Fungi Native from a Mediterranean Saline Area Enhance Maize Tolerance to Salinity through Improved Ion Homeostasis. Plant Cell Environ..

[132] Wang H., An T., Huang D., Liu R., Xu B., Zhang S., Deng X., Siddique K.H.M., Chen Y. (2021). Arbuscular Mycorrhizal Symbioses Alleviating Salt Stress in Maize Is Associated with a Decline in Root-to-Leaf Gradient of Na^+^/K^+^ Ratio. BMC Plant Biol..

[133] Liu F., Xu Y., Han G., Wang W., Li X., Cheng B. (2018). Identification and Functional Characterization of a Maize Phosphate Transporter Induced by Mycorrhiza Formation. Plant Cell Physiol..

[134] Ferrol N., Azcón-Aguilar C., Pérez-Tienda J. (2019). Review: Arbuscular Mycorrhizas as Key Players in Sustainable Plant Phosphorus Acquisition: An Overview on the Mechanisms Involved. Plant Sci..

[135] Yang S.Y., Grønlund M., Jakobsen I., Grotemeyer M.S., Rentsch D., Miyao A., Hirochika H., Kumar C.S., Sundaresan V., Salamin N. (2012). Nonredundant Regulation of Rice Arbuscular Mycorrhizal Symbiosis by Two Members of the *PHOSPHATE TRANSPORTER1* Gene Family. Plant Cell.

[136] Liu F., Xu Y., Jiang H., Jiang C., Du Y., Gong C., Wang W., Zhu S., Han G., Cheng B. (2016). Systematic Identification, Evolution and Expression Analysis of the *Zea mays PHT1* Gene Family Reveals Several New Members Involved in Root Colonization by Arbuscular Mycorrhizal Fungi. Int. J. Mol. Sci..

[137] Xie X., Huang W., Liu F., Tang N., Liu Y., Lin H., Zhao B. (2013). Functional Analysis of the Novel Mycorrhiza-Specific Phosphate Transporter AsPT1 and PHT1 Family from *Astragalus sinicus* during the Arbuscular Mycorrhizal Symbiosis. New Phytol..

[138] Wang D., Dong W., Murray J., Wang E. (2022). Innovation and Appropriation in Mycorrhizal and Rhizobial Symbioses. Plant Cell.

[139] Walder F., Brulé D., Koegel S., Wiemken A., Boller T., Courty P.E. (2015). Plant Phosphorus Acquisition in a Common Mycorrhizal Network: Regulation of Phosphate Transporter Genes of the *Pht1* Family in Sorghum and Flax. New Phytol..

[140] Liu L., Li J., Yue F., Yan X., Wang F., Bloszies S., Wang Y. (2018). Effects of arbuscular mycorrhizal inoculation and biochar amendment on maize growth, cadmium uptake and soil cadmium speciation in Cd-contaminated soil. Chemosphere.

[141] Gu L., Zhao M., Ge M., Zhu S., Cheng B., Li X. (2019). Transcriptome Analysis Reveals Comprehensive Responses to Cadmium Stress in Maize Inoculated with Arbuscular Mycorrhizal Fungi. Ecotoxicol. Environ. Saf..

[142] Zhang B., Shi F., Zheng X., Pan H., Wen Y., Song F. (2023). Effects of AMF Compound Inoculants on Growth, Ion Homeostasis, and Salt Tolerance-Related Gene Expression in *Oryza sativa* L. Under Salt Treatments. Rice.

[143] Olalde-Portugal V., Cabrera-Ponce J.L., Gastelum-Arellanez A., Guerrero-Rangel A., Winkler R., Valdés-Rodríguez S. (2020). Proteomic Analysis and Interactions Network in Leaves of Mycorrhizal and Nonmycorrhizal Sorghum Plants under Water Deficit. PeerJ.

[144] Puccio G., Ingraffia R., Mercati F., Amato G., Giambalvo D., Martinelli F., Sunseri F., Frenda A.S. (2023). Transcriptome Changes Induced by Arbuscular Mycorrhizal Symbiosis in Leaves of Durum Wheat (*Triticum durum* Desf.) Promote Higher Salt Tolerance. Sci. Rep..

[145] El-Sawah A.M., Abdel-Fattah G.G., Holford P., Korany S.M., Alsherif E.A., AbdElgawad H., Ulhassan Z., Jośko I., Ali B., Sheteiwy M.S. (2023). *Funneliformis constrictum* Modulates Polyamine Metabolism to Enhance Tolerance of *Zea mays* L. to Salinity. Microbiol. Res..

[146] Li S., Yang W., Guo J., Li X., Lin J., Zhu X. (2020). Changes in Photosynthesis and Respiratory Metabolism of Maize Seedlings Growing under Low Temperature Stress May Be Regulated by Arbuscular Mycorrhizal Fungi. Plant Physiol. Biochem..

[147] Kosakivska I.V., Vedenicheva N.P., Babenko L.M., Voytenko L.V., Romanenko K.O., Vasyuk V.A. (2022). Exogenous Phytohormones in the Regulation of Growth and Development of Cereals under Abiotic Stresses. Mol. Biol. Rep..

[148] Ge J., Li D., Ding J., Xiao X., Liang Y. (2023). Microbial Coexistence in the Rhizosphere and the Promotion of Plant Stress Resistance: A Review. Environ. Res..

[149] Jung S.C., Martinez-Medina A., Lopez-Raez J.A., Pozo M.J. (2012). Mycorrhiza-Induced Resistance and Priming of Plant Defenses. J. Chem. Ecol..

[150] Zipfel C. (2008). Pattern-Recognition Receptors in Plant Innate Immunity. Curr. Opin. Immunol..

[151] Boller T., Felix G. (2009). A Renaissance of Elicitors: Perception of Microbe-Associated Molecular Patterns and Danger Signals by Pattern-Recognition Receptors. Annu. Rev. Plant Biol..

[152] Hohmann P., Messmer M.M. (2017). Breeding for Mycorrhizal Symbiosis: Focus on Disease Resistance. Euphytica.

[153] Li M., Wang R., Tian H., Gao Y. (2018). Transcriptome Responses in Wheat Roots to Colonization by the Arbuscular Mycorrhizal Fungus *Rhizophagus irregularis*. Mycorrhiza.

[154] Campos-Soriano L., García-Martínez J., Segundo B.S. (2012). The Arbuscular Mycorrhizal Symbiosis Promotes the Systemic Induction of Regulatory Defence-related Genes in Rice Leaves and Confers Resistance to Pathogen Infection. Mol. Plant Pathol..

[155] Sousa T.P.D., Chaibub A.A., Silva G.B.D., Filippi M.C.C.D. (2020). *Trichoderma asperellum* Modulates Defense Genes and Potentiates Gas Exchanges in Upland Rice Plants. Physiol. Mol. Plant Pathol..

[156] El-Sharkawy H.H.A., Rashad Y.M., Elazab N.T. (2023). Induction of Multiple Defense Responses in Wheat Plants against Stripe Rust Using Mycorrhizal Fungi and HH1. BioControl.

[157] Anjali, Kumar S., Korra T., Thakur R., Arutselvan R., Kashyap A.S., Nehela Y., Chaplygin V., Minkina T., Keswani C. (2023). Role of Plant Secondary Metabolites in Defence and Transcriptional Regulation in Response to Biotic Stress. Plant Stress.

[158] Rashad Y.M., El-Sharkawy H.H., Abdalla S.A., Ibrahim O.M., Elazab N.T. (2023). Mycorrhizal Colonization and *Streptomyces viridosporus* HH1 Synergistically Up-Regulate the Polyphenol Biosynthesis Genes in Wheat against Stripe Rust. BMC Plant Biol..

[159] Recchia G.H., Konzen E.R., Cassieri F., Caldas D.G.G., Tsai S.M. (2018). Arbuscular Mycorrhizal Symbiosis Leads to Differential Regulation of Drought-Responsive Genes in Tissue-Specific Root Cells of Common Bean. Front. Microbiol..

[160] Vangelisti A., Natali L., Bernardi R., Sbrana C., Hassani-pak K., Hughes D., Cavallini A., Giovannetti M. (2018). Transcriptome Changes Induced by Arbuscular Mycorrhizal Fungi in Sunflower (*Helianthus annuus* L.) Roots. Sci. Rep..

[161] Siddique I., Shah T., Ali A., Ahmad I., Roberto D., Munsif F. (2022). Arbuscular Mycorrhizal Fungi and Biofilm Forming Bacteria Act Synergistically to Modulate Proline Metabolism, Antioxidant Defense System and Aquaporin Genes Expression Under Drought Stress. Preprints.

[162] Singh R.K., Shweta S., Muthamilarasan M., Rani R., Prasad M. (2019). Study on Aquaporins of *Setaria italica* Suggests the Involvement of SiPIP3;1 and SiSIP1;1 in Abiotic Stress Response. Funct. Integr. Genom..

[163] Kabir A.H., Debnath T., Das U., Prity S.A., Haque A., Rahman M.M., Parvez M.S. (2020). Arbuscular Mycorrhizal Fungi Alleviate Fe-Deficiency Symptoms in Sunflower by Increasing Iron Uptake and Its Availability along with Antioxidant Defense. Plant Physiol. Biochem..

[164] Liao Q., Zhou T., Yao J., Han Q., Song H., Guan C., Hua Y., Zhang Z. (2018). Genome-Scale Characterization of the *Vacuole Nitrate Transporter Chloride Channel* (*CLC*) Genes and Their Transcriptional Responses to Diverse Nutrient Stresses in Allotetraploid Rapeseed. PLoS ONE.

[165] Abbaspour H., Pour F.S.N., Abdel-Wahhab M.A. (2021). Arbuscular Mycorrhizal Symbiosis Regulates the Physiological Responses, Ion Distribution and Relevant Gene Expression to Trigger Salt Stress Tolerance in Pistachio. Physiol. Mol. Biol. Plants.

[166] Salloum M.S., Insani M., Monteoliva M.I., Menduni M.F., Silvente S., Carrari F., Luna C. (2019). Metabolic Responses to Arbuscular Mycorrhizal Fungi Are Shifted in Roots of Contrasting Soybean Genotypes. Mycorrhiza.

[167] You J., Zhang Y., Liu A., Li D., Wang X., Dossa K., Zhou R., Yu J., Zhang Y., Wang L. (2019). Transcriptomic and Metabolomic Profiling of Drought-Tolerant and Susceptible Sesame Genotypes in Response to Drought Stress. BMC Plant Biol..

[168] Shimoia E.P., Da-Silva C.J., Posso D.A., Martins T.d.S., Agualongo D.A.P., de Oliveira A.C.B., Amarante L.D. (2023). Co-inoculation of Seeds with *Bradyrhizobium*, *Azospirillum*, and *Rhizophagus* Improves Nitrogen Assimilation and Growth in Soybean Plants Subjected to Waterlogging. Russ. J. Plant Physiol..

[169] Zhao M.-J., Yin L.-J., Liu Y., Ma J., Zheng J.-C., Lan J.-H., Fu J.-D., Chen M., Xu Z.-S., Ma Y.-Z. (2019). The ABA-Induced Soybean *ERF* Transcription Factor Gene *GmERF75* Plays a Role in Enhancing Osmotic Stress Tolerance in Arabidopsis and Soybean. BMC Plant Biol..

[170] Spagnoletti F.N., Leiva M., Chiocchio V., Lavado R.S. (2018). Phosphorus Fertilization Reduces the Severity of Charcoal Rot (*Macrophomina phaseolina*) and the Arbuscular Mycorrhizal Protection in Soybean. J. Plant Nutr. Soil Sci..

[171] Slimani A., Oufdou K., Meddich A. (2023). Intercropping with Alfalfa and Co-Inoculation of AMF and PGPR Improve Growth, Yield, Grain Bioactive Quality, and Soil Fertility of Barley. Arch. Agron. Soil Sci..

[172] Nafady N.A., Hashem M., Hassan E.A., Ahmed H.A.M., Alamri S.A. (2019). The Combined Effect of Arbuscular Mycorrhizae and Plant-Growth-Promoting Yeast Improves Sunflower Defense against *Macrophomina phaseolina* Diseases. Biol. Control.

[173] Elewa I.S., Mostafa M.H., Sahab A.F., Ziedan E.H. (2011). Direct Effect of Biocontrol Agents on Wilt and Root-Rot Diseases of Sesame. Arch. Phytopathol. Plant Prot..

[174] Rashad Y., Aseel D., Hammad S., Elkelish A. (2020). *Rhizophagus irregularis* and *Rhizoctonia solani* Differentially Elicit Systemic Transcriptional Expression of Polyphenol Biosynthetic Pathways Genes in Sunflower. Biomolecules.

[175] Ahmed W.K., Alsalim H.A.A., Mohammed A.T., Youssef H.M. (2023). Evaluation of the Effectiveness of Some Mycorrhizal Fungi Isolates against Charcoal Rot Disease. Egypt. J. Biol. Pest Control.

[176] Malik R.J., Dixon M.H., Bever J.D. (2016). Mycorrhizal Composition Can Predict Foliar Pathogen Colonization in Soybean. Biol. Control..

[177] Gao X., Lu X., Wu M., Zhang H., Pan R., Tian J., Li S., Liao H. (2012). Co-Inoculation with Rhizobia and AMF Inhibited Soybean Red Crown Rot: From Field Study to Plant Defense-Related Gene Expression Analysis. PLoS ONE.

[178] Aquino B., Bradley J.M., Lumba S. (2021). On the Outside Looking in: Roles of Endogenous and Exogenous Strigolactones. Plant J..

[179] Clark J., Bennett T. (2023). Cracking the Enigma: Understanding Strigolactone Signalling in the Rhizosphere. J. Exp. Bot..

[180] Weng W., Yan J., Zhou M., Yao X., Gao A., Ma C., Cheng J., Ruan J. (2022). Roles of Arbuscular Mycorrhizal Fungi as a Biocontrol Agent in the Control of Plant Diseases. Microorganisms.

[181] Liu F., Rice J.H., Lopes V., Grewal P., Lebeis S.L., Hewezi T., Staton M.E. (2020). Overexpression of Strigolactone-Associated Genes Exerts Fine-Tuning Selection on Soybean Rhizosphere Bacterial and Fungal Microbiome. Phytobiomes J..

[182] Belmondo S., Marschall R., Tudzynski P., López Ráez J.A., Artuso E., Prandi C., Lanfranco L. (2017). Identification of Genes Involved in Fungal Responses to Strigolactones Using Mutants from Fungal Pathogens. Curr. Genet..

[183] Dor E., Joel D.M., Kapulnik Y., Koltai H., Hershenhorn J. (2011). The Synthetic Strigolactone GR24 Influences the Growth Pattern of Phytopathogenic Fungi. Planta.

[184] Foo E., Blake S.N., Fisher B.J., Smith J.A., Reid J.B. (2016). The Role of Strigolactones during Plant Interactions with the Pathogenic Fungus *Fusarium oxysporum*. Planta.

[185] Fiorilli V., Vallino M., Biselli C., Faccio A., Bagnaresi P., Bonfante P. (2015). Host and Non-Host Roots in Rice: Cellular and Molecular Approaches Reveal Differential Responses to Arbuscular Mycorrhizal Fungi. Front. Plant Sci..

[186] Guo R., Wu Y., Liu C., Liu Y., Tian L., Cheng J., Pan Z., Wang D., Wang B. (2022). OsADK1, a Novel Kinase Regulating Arbuscular Mycorrhizal Symbiosis in Rice. New Phytol..

[187] Guo N., Li L., Cui J., Cai B. (2021). Effects of *Funneliformis mosseae* on the Fungal Community in and Soil Properties of a Continuously Cropped Soybean System. Appl. Soil Ecol..

[188] Pozo M.J., Azcón-Aguilar C. (2007). Unraveling Mycorrhiza-Induced Resistance. Curr. Opin. Plant Biol..

[189] Boller T., He S.Y. (2009). Innate Immunity in Plants: An Arms Race Between Pattern Recognition Receptors in Plants and Effectors in Microbial Pathogens. Science.

[190] Thomma B.P.H.J., Nürnberger T., Joosten M.H.A.J. (2011). Of PAMPs and Effectors: The Blurred PTI-ETI Dichotomy. Plant Cell.

[191] Nishad R., Ahmed T., Rahman V.J., Kareem A. (2020). Modulation of Plant Defense System in Response to Microbial Interactions. Front. Microbiol..

[192] Oldroyd G.E.D. (2013). Speak, Friend, and Enter: Signalling Systems That Promote Beneficial Symbiotic Associations in Plants. Nat. Rev. Microbiol..

[193] Lapin D., Van Den Ackerveken G. (2013). Susceptibility to Plant Disease: More than a Failure of Host Immunity. Trends Plant Sci..

[194] Cameron D.D., Neal A.L., Van Wees S.C.M., Ton J. (2013). Mycorrhiza-Induced Resistance: More than the Sum of Its Parts?. Trends Plant Sci..

[195] Pieterse C.M.J., Van Der Does D., Zamioudis C., Leon-Reyes A., Van Wees S.C.M. (2012). Hormonal Modulation of Plant Immunity. Annu. Rev. Cell Dev. Biol..

[196] Song F., Song G., Dong A., Kong X. (2011). Regulatory Mechanisms of Host Plant Defense Responses to Arbuscular Mycorrhiza. Acta Ecol. Sin..

[197] Siciliano V., Genre A., Balestrini R., deWit P.J.G.M., Bonfante P. (2007). Pre-Penetration Apparatus Formation During AM Infection Is Associated with a Specific Transcriptome Response in Epidermal Cells. Plant Signal. Behav..

[198] Bai L., Sun H.-B., Liang R.-T., Cai B.-Y. (2019). iTRAQ Proteomic Analysis of Continuously Cropped Soybean Root Inoculated With *Funneliformis mosseae*. Front. Microbiol..

